# Evolution of Sequence Type 4821 Clonal Complex Hyperinvasive and Quinolone-Resistant Meningococci 

**DOI:** 10.3201/eid2704.203612

**Published:** 2021-04

**Authors:** Mingliang Chen, Odile B. Harrison, Holly B. Bratcher, Zhiyan Bo, Keith A. Jolley, Charlene M.C. Rodrigues, James E. Bray, Qinglan Guo, Xi Zhang, Min Chen, Martin C.J. Maiden

**Affiliations:** Shanghai Municipal Center for Disease Control and Prevention, Shanghai, China (M. Chen, X. Zhang, M. Chen);; University of Oxford, Oxford, UK (O.B. Harrison, H.B. Bratcher, Z. Bo, K.A. Jolley, C.M.C. Rodrigues, J.E. Bray, M.C.J. Maiden);; Fudan University, Huashan Hospital, Shanghai (Q. Guo)

**Keywords:** *Neisseria meningitidis*, meningitis/encephalitis, ST4821 clonal complex, bacteria, genome, phylogenetic analysis, recombination, quinolone resistance, antimicrobial resistance, China

## Abstract

Expansion of quinolone-resistant *Neisseria meningitidis* clone China^CC4821-R1-C/B^ from sequence type (ST) 4821 clonal complex (CC4821) caused a serogroup shift from serogroup A to serogroup C invasive meningococcal disease (IMD) in China. To determine the relationship among globally distributed CC4821 meningococci, we analyzed whole-genome sequence data from 173 CC4821 meningococci isolated from 4 continents during 1972–2019. These meningococci clustered into 4 sublineages (1–4); sublineage 1 primarily comprised of IMD isolates (41/50, 82%). Most isolates from outside China (40/49, 81.6%) formed a distinct sublineage, the Europe–USA cluster, with the typical strain designation B:P1.17-6,23:F3-36:ST-3200(CC4821), harboring mutations in penicillin-binding protein 2. These data show that the quinolone-resistant clone China^CC4821-R1-C/B^ has expanded to other countries. The increasing distribution worldwide of serogroup B CC4821 raises the concern that CC4821 has the potential to cause a pandemic that would be challenging to control, despite indirect evidence that the Trumenba vaccine might afford some protection.

*Neisseria meningitidis*, a leading cause of bacterial meningitis and septicemia globally, causes ≈1.2 million invasive meningococcal disease (IMD) cases annually and a case-fatality rate of 11% (*1*). Meningococci are classified into 12 serogroups based on capsular polysaccharides (*1*); genetic relationships among isolates are defined by clonal complexes (CCs) identified by multilocus sequence typing (MLST), which are surrogates for lineages (*2*). The relationship among serogroups, CCs (lineages), and IMD fluctuates over time and by location, but IMD isolates are dominated by CCs known as hyperinvasive lineages, usually associated with one of the 6 disease-causing serogroups (MenA, MenB, MenC, MenW, MenX, and MenY).

In China, the national dissemination of hyperinvasive sequence type (ST) 4821 clonal complex (CC4821) meningococci led to a shift in IMD epidemiology from mostly MenA to predominantly MenC (*3*,*4*). Although no quinolone resistance was identified in CC4821 in China during 1965–1985, high-frequency resistance (79%) occurred from 2005 onward due to expansion of the quinolone-resistant clone China^CC4821-R1-C/B^ (*5*). Previous studies discovered that CC4821 can be divided into 2 groups, with group 1 associated with IMD (*6*,*7*). Peng et al. identified 6 strain-specific genome regions resulting from horizontal gene transfer (HGT) in isolate 053442 (*8*); this finding was consistent with the emergence of the China^CC4821-R1-C/B^ clone associated with multiple HGT events within genes encoding surface antigens (*6*), although the donors of these events were not identified.

Globally, the number of CC4821 IMD isolates has increased. At the time CC4821 was identified, isolates were confined to China (*4*,*9*); however, by June 2020, a total of 59 CC4821 isolates had been identified in 19 countries worldwide (Figure 1). Moreover, 3 IMD cases caused by quinolone-resistant CC4821 isolates were reported in Canada (n = 2) and Japan (n = 1) after 2013 (*10*,*11*); 3 other CC4821 isolates were found to colonize the anorectal tract of men who have sex with men (MSM) (*12*). We investigated the genomic events leading to the emergence and expansion of hyperinvasive CC4821 meningococci by describing the phylogenetic relationships among meningococci with different serogroups (MenC, MenB, MenW, and nongroupable), sources (IMD, carriage, and MSM), locations (China or other countries), and dates of isolation (1972–1978 vs. 2004–2019). We assessed genes encoding key antigens and antimicrobial resistance phenotypes, identified putative donors of HGT events unique to the epidemic and quinolone-resistant clone China^CC4821-R1-C/B^, and characterized isolates outside of China.

**Figure 1:**
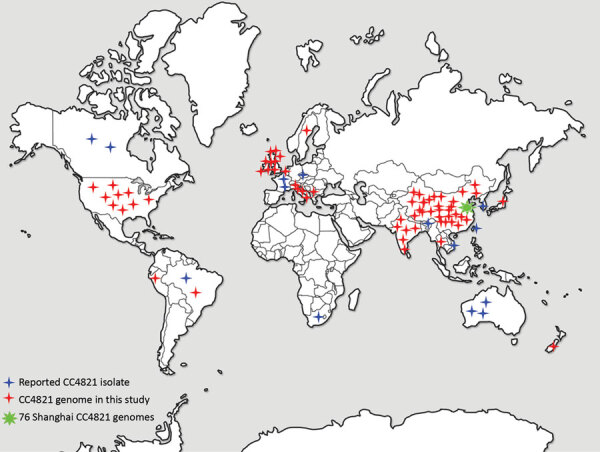
Distribution of CC4821 *Neisseria meningitidis* isolates worldwide. CC4821 isolates were identified in China and in 19 countries of Europe, Africa, North America, South America, Oceania, and Asia. CC, clonal complex.

## Materials and Methods

### Isolate Collection and Whole-Genome Sequencing

A total of 173 CC4821 genomes were collected dating from 1972–1978 (n = 19) and 2004–2019 (n = 154), including isolates from IMD (66/173, 38.2%), genitourinary sites (6/173, 3.5%), asymptomatic carriage (86/173, 49.7%), and unknown sources (15/173, 8.7%) (Appendix 1 Table 1). Shanghai CDC sequenced 76 CC4821 isolates with Illumina HiSeq (Illumina, https://www.illumina.com) using paired-end 150 base reads as previously described (*13*). An additional 97 publicly available CC4821 genomes consisted of 48 genomes from 14 provinces of China, including the reference strain 053442 (*6*–*8*) and 49 genomes from countries outside of China, including the United Kingdom (n = 20), United States (n = 8), and 11 other countries (n = 21) (Figure 1; Appendix 1 Table 1) (*10*,*12*,*14*–*17*). The completeness and contamination of the genomes were evaluated using CheckM (*18*).

### Antigenic and Antimicrobial Resistance Characteristics of CC4821 Genomes

To describe the antigenic and antimicrobial resistance characteristics of CC4821 genomes, we extracted from genomes nucleotides of 9 antigen coding genes (*porA*, *fHbp*, *nhba*, *porB*, *fetA*, *opcA*, *nspA*, *tbpA*, and *NMB0315*) (*19*–*22*) and 5 resistance-associated genes (*gyrA*, *parC*, *penA*, *ponA*, and *rpoB*) (*23*,*24*) for analysis. We annotated and analyzed deduced encoding factor H–binding protein (fHbp), *Neisseria* heparin-binding antigen (NHBA), *Neisseria* adhesion antigen (NadA), and outer membrane protein (PorA) peptides and deduced meningococcal vaccine antigen reactivity (MenDeVAR) index from the PubMLST *Neisseria* database (*25*).

### Identifying CC4821 (L44) Sublineages

In the *Neisseria* PubMLST database, a lineage-specific core genome MLST typing scheme containing loci found in 95% of CC4821 isolates was established and designated L44 cgMLST consistent with the previously described CC4821 lineage 44 (*26*). We compared the 173 CC4821 genomes using Genome Comparator (*27*) and the L44 cgMLST scheme, identifying distinct sublineages. To characterize each sublineage, we visualized a FASTA output from the Genome Comparator Tool using all 2,860 defined loci (NEIS0001–NEIS3173, not contiguous) using MEGA version 5 (*28*). We used Z2491 (GenBank accession no. NC_003116) as outgroup in accordance with previous studies (*6*,*8*). Assembled contigs and annotation information of 173 genomes in this study can be accessed at https://pubMLST.org/neisseria (Appendix 1 Table 1).

### Identifying and Characterizing Unique Alleles in Sublineages

We determined shared and unique alleles using outputs from Genome Comparator. An allele was defined as unique to a sublineage if it was present in >90% of the genomes in that sublineage but absent in other sublineages. Genes with unique alleles were functionally characterized according to the Kyoto Encyclopedia of Genes and Genomes Orthology groupings of its database (*29*).

### Identifying HGT Events and Putative Donors

Inputting the aligned sequences generated from Parsnp (*30*), we predicted putative HGT events using Gubbins (*31*). To search for potential donors, we blasted alleles and sequences of contiguous loci that were predicted to originate from HGT against the PubMLST database. We identified potential donors as previously described (*32*). We labeled recombination areas with unique loci on the circular genome map of genome 053442 by BLAST (https://blast.ncbi.nlm.nih.gov/Blast.cgi) comparisons to strains of other sublineages, as generated using BRIG (*33*).

### Screening Molecular Markers of MSM Infection Strains from Europe

In addition to the lineage of 11.2 possessing PorA P1.5–1,10–8, 3 other molecular features have been identified in meningococci causing infections among MSM in Europe during 2012–2014; these features were functional nitrite reductase (AniA); frameshifted *fHbp* allele found mostly in urethritis and proctitis isolates; and *penA327* that had reduced susceptibility to penicillin and third-generation cephalosporins (*34*). These 3 molecular markers were screened among all the 173 CC4821 genomes.

## Results

### Isolate Characterization

The 173 CC4821 isolates represented 46 different STs; ST4821 (n = 41, 23.7%) and ST3200 (n = 30, 17.3%) were the most prevalent. We identified 43 PorA subtypes, of which P1.7-2,14 (n = 25, 14.5%) and P1.17-6,23 (n = 18, 10.4%) were the most frequent. We identified 27 FetA variants; F3-3 (n = 47, 27.2%) and F3-36 (n = 37, 21.4%) were the most prevalent (Appendix 1 Table 1).

### Identifying 4 Sublineages

**Figure 2:**
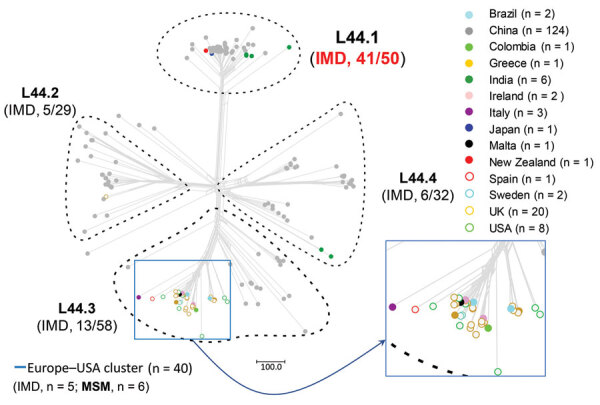
Allele-based sublineages of clonal complex 4821 *Neisseria meningitidis* identified using lineage 44 core genome multilocus sequence typing scheme. The inset shows the country distribution of the 40 genomes constituting the Europe–USA cluster. IMD, invasive meningococcal disease; MSM, men who have sex with men.

**Figure 3:**
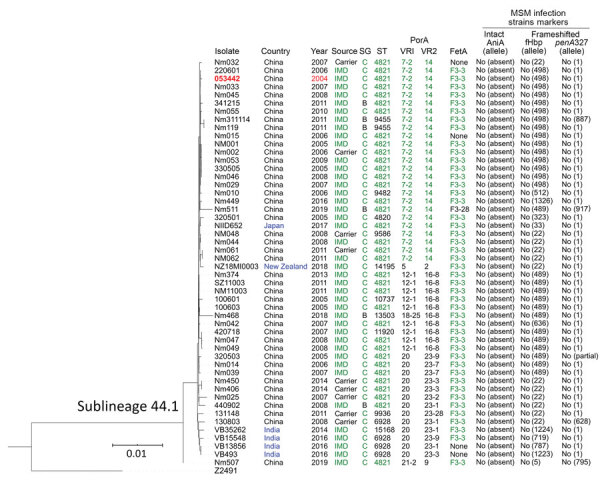
Phylogenetic tree and data of clonal complex 4821 *Neisseria meningitidis* sublineage L44.1 (China^CC4821-R1-C/B^) isolates. Red text indicates the oldest isolate of the sublineage; blue text, the isolates from countries outside of China; and green text, the dominant type or allele. Scale bar indicates substitutions per site. IMD, invasive meningococcal disease; MSM, men who have sex with men; SG, serogroup; ST, sequence type; VR, variable region.

We identified 2,161 loci in reference genome 053442, including 1,699 core genes. Most (1,527/1,699, 89.9%) of the core loci had *p*-distance values of 0–0.1; 0.8% (14/1,699) showed high *p*-distance values of 0.50–0.68. On the basis of the L44 cgMLST scheme, we divided the CC4821 isolates into 4 sublineages (Figure 2): L44.1, identical to the China^CC4821-R1-C/B^ clone (n = 50, 28.9%), composed of isolates from China (n = 44) and other countries (n = 6) during 2004–2019 that were very closely related (Figure 3); L44.2 (n = 29, 16.8%), composed of isolates from China (n = 28) and the United Kingdom (n = 1) during 2005–2019; L44.3 (n = 58, 33.5%), composed of isolates from China (n = 18) and countries outside China (n = 40) during 1977–2019; and L44.4 (n = 32, 18.5%), composed of isolates from China (n = 30) and India (n = 2) during 1972–2017. Four additional isolates from China were not assigned to any sublineages.

### Features of the 4 Sublineages

The percentage of IMD isolates was significantly higher in L44.1 (41/50, 82%) than the other 3 sublineages (17.2%–22.4%; p<0.001) (Figure 2). L44.1, containing the reference strain 053442, was mainly composed of MenC isolates (44/50, 88%) and had ST4821 as its central ST. L44.2, was mainly composed of MenB isolates (27/29, 93.1%) and its central ST was ST5664. L44.3 was mainly composed of MenB (55/58, 94.8%) with ST3200 as its central ST. L44.4 was mainly composed of MenC (14/32, 43.8%) and MenW (11/32, 34.4%) with its central ST3436 (Appendix 2 Figure 1). 

**Figure 4:**
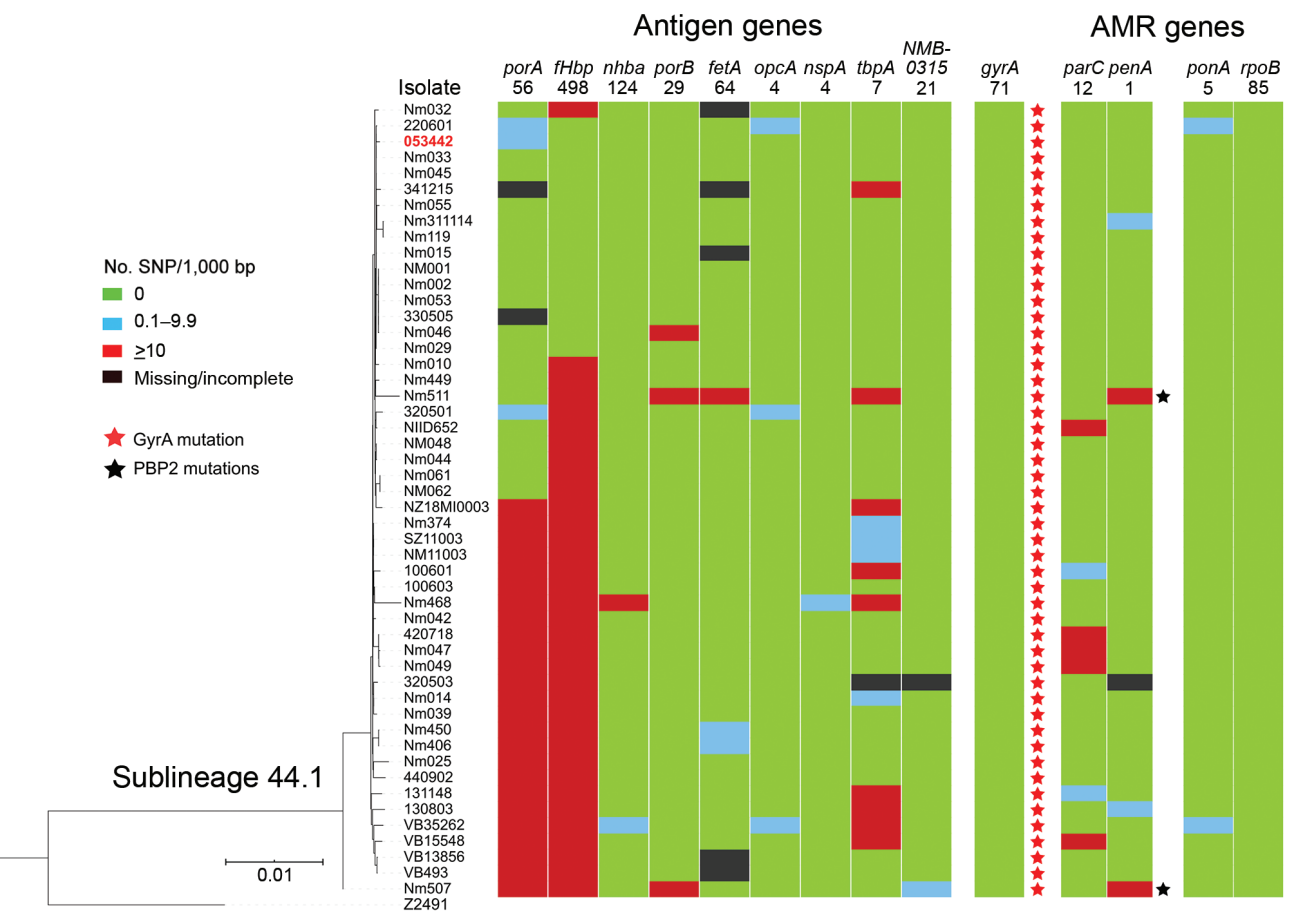
Genomic diversity of clonal complex 4821 *Neisseria meningitidis* sublineage L44.1 (China^CC4821-R1-C/B^) isolates. The numbers underneath the antigen genes and AMR genes are the dominant alleles for that particular gene, and the colored blocks for SNPs/1,000 bp were determined using the allele number labeled above each column as the reference allele. AMR, antimicrobial resistance; SNP, single-nucleotide polymorphism.

**Figure 5:**
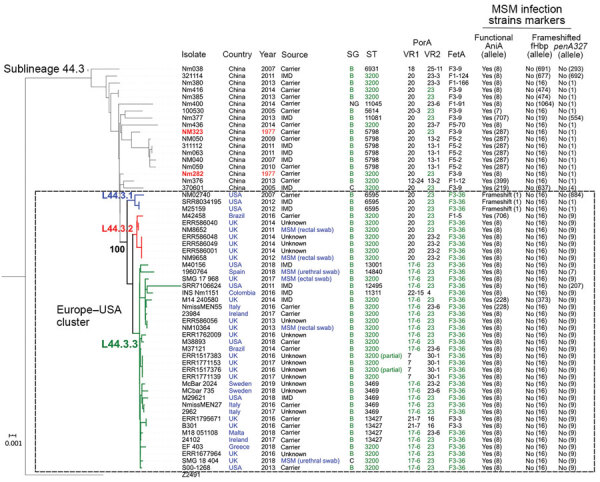
Phylogenetic tree and data of clonal complex 4821 *Neisseria meningitidis* sublineage L44.3 isolates. Red text indicates the oldest isolates of the sublineage; blue text, the isolates from countries outside of China and the isolates from genitourinary swabs from MSM; and green text, the dominant type or allele. The Europe–USA cluster can be further divided into 3 subclusters: subcluster L44.3.1, composed of 3 ST6595 isolates from the United States, all of which contained putatively nonfunctional AniA; L44.3.2, composed of 7 ST3200 isolates from the United Kingdom (n = 6) and Brazil (n = 1); and L44.3.3, composed of 30 isolates with multiple geographic locations. All the isolates from urethral (n = 2) and rectal (n = 4) swabs were assigned to L44.3.2 and L44.3.3, both of which comprised isolates with putatively functional AniA. Scale bar indicates substitutions per site. IMD, invasive meningococcal disease; MSM, men who have sex with men; NG, nongroupable; SG, serogroup; ST, sequence type; VR, variable region.

**Figure 6:**
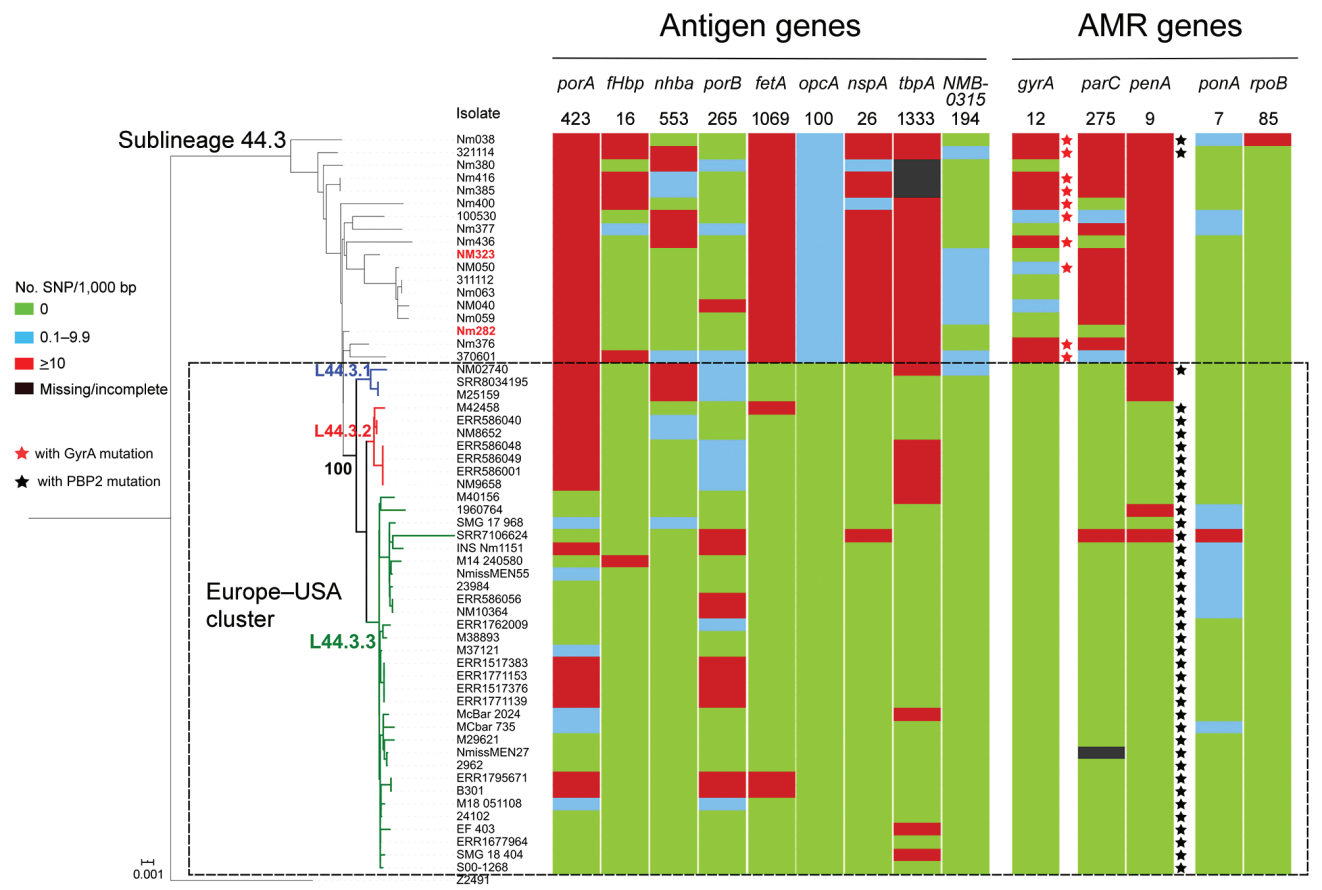
Genomic diversity of clonal complex 4821 *Neisseria meningitidis* sublineage L44.3 isolates. The numbers underneath the antigen genes and AMR genes are the dominant alleles for that particular gene, and the color blocks for SNPs/1,000 bp were determined using the allele number labeled above each column as the reference allele. The Europe–USA cluster can be further divided into 3 subclusters: subcluster L44.3.1, composed of 3 ST6595 isolates from the United States, all of which contained putatively nonfunctional AniA; L44.3.2, composed of 7 ST3200 isolates from the United Kingdom (n = 6) and Brazil (n = 1); and L44.3.3, composed of 30 isolates with multiple geographic locations. All the isolates from urethral (n = 2) and rectal (n = 4) swabs were assigned to L44.3.2 and L44.3.3, both of which comprised isolates with putatively functional AniA. Scale bar indicates substitutions per site. AMR, antimicrobial resistance; SNP, single-nucleotide polymorphism.

Analysis of the 5 antimicrobial resistance genes revealed that both *gyrA*-71 (with T91I) and *parC*-12 were specific to L44.1; *parC*-275 and *penA*-9 (with 5 mutations) were both specific to L44.3, and *gyrA*-294 (with T91I) was discovered only in L44.4 (Table 1; Appendix 2 Figures 2–6). In L44.1, all of the isolates possessed the quinolone resistance–associated mutation T91I in GyrA (Figure 4). In L44.3, 40/58 (69.0%) harbored PBP2 mutations, almost always from countries outside of China (38/40, 95%) (Figures 5, 6).

**Table 1:** Specific alleles of antimicrobial resistance genes in 4 sublineages of clonal complex 4821 of *Neisseria meningitidis*

Sublineage	Resistant, allele no. (no. isolates)				
	*gyrA*	*parC*	*penA*	*ponA*	*rpoB*
L44.1, n = 50	71 (50)	12 (43)	None	None	None
L44.2, n = 29	None	None	None	None	None
L44.3, n = 58	None	275 (41)	9 (35)	None	None
L44.4, n = 32	294 (11)	None	None	None	None

### Vaccine Antigens among the 4 Sublineages

**Figure 7:**
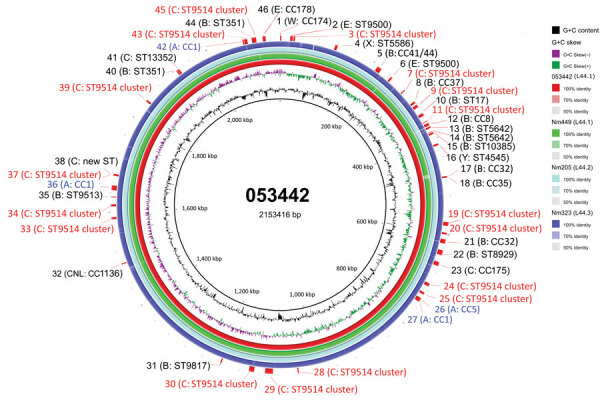
Circular genome map of CC4821 *Neisseria meningitidis* genome 053442 with BLAST (https://blast.ncbi.nlm.nih.gov/Blast.cgi) comparisons to the genomes of other sublineages. The innermost rings show guanine and cytosine (G+C) content (black) and G+C skew (negative in purple, positive in green) of genome 053442. The 4 outer rings show BLAST comparisons (using BLASTn and an E-value cutoff of 10.0) to the complete genome sequence of 053442 (red), Nm449 (green), Nm205 (pale blue), and Nm323 (blue); shading on rings indicates percentage identity as indicated in the key. Labels around the outer ring refer to the 46 HGT events involving 149 unique loci that are labeled with their possible donor strain. Red text indicates loci related to most common donors; blue text indicates those with serogroup A lineage donors. CC, clonal complex; HGT, horizontal gene transfer; Nm, *N. meningitidis*; ST, sequence type.

Analysis of 9 antigenic genes identified several alleles unique to a certain sublineage (Table 2; Appendix 2 Figures 7–17). For example, FetA-VR F3-3 was found in L44.1, F1-91 in L44.2, F3-36 and F3-9 in L44.3, and F1-7 in L44.4 isolates (Appendix 2 Figure 11). In L44.1, most isolates had the same antigenic gene profile (*nhba*-124, *porB*-29, *fetA*-64, *opcA*-4, *nspA*-4, *tbpA*-7, and *NMB0315*-21) (Figure 4), and 25/50 (50%) had the PorA subtype of P1.7-2,14 (Figure 3). In L44.3, most had the same gene profile (*fHbp*-16, *nhba*-553, *porB*-265, *fetA*-1069, *opcA*-100, *nspA*-26, and *NMB0315*-194), with *porA* and *tbpA* showing high genetic diversity (Figure 5).

**Table 2:** Specific alleles of antigenic genes in 4 sublineages of clonal complex 4821 of *Neisseria meningitidis*

Sublineage	Antigen allele no. (no. isolates)								
	PorA	*fHbp*	*nhba*	PorB	FetA-VR	*opcA*	*nspA*	*tbpA*	*NMB-0315*
L44.1, n = 50	P1.7–2,14 (25)	22 (12)	124 (48)	3–48 (47)	F3–3 (45)	None	4 (49)	7 (36)	None
	P1.12–1,16–8 (9)	489 (13)							
		498 (15)							
L44.2, n = 29	None	None	965 (10)	3–81 (15)	F1–91 (20)	None	None	None	335 (26)
			967 (12)						
L44.3, n = 58	P1.17–6,23-x* (23)	None	None	3–229 (35)	F3–9 (8)	100 (40)	26 (39)	1,333 (31)	194 (49)
					F3–36 (37)				
L44.4, n = 32	P1.5–3,10–2 (8)	None	None	3–460 (7)	F1–7 (11)	None	117 (20)	None	None

*23-x refers to 23, 23–2, and 23–6.

We analyzed deduced peptide sequences for vaccine antigen constituents among MenB isolates (n = 97). We identified 16 fHbp peptides, of which peptide 16 (variant 2/subfamily A) was present in 70/97 (72.2%) isolates, including 31/70 isolates from China. There were 20 NHBA peptides, of which alleles 669 (46/95, 48.4%), 901 (11/95, 11.6%), and 668 (10/95, 10.5%) occurred most frequently. The *nadA* gene was absent in all isolates (including other serogroups). Of 31 PorA VR1/VR2 combinations, the most frequently occurring was P1.20,23 (11/97, 11.3%).

MenDeVAR Index values were assigned for MenB disease isolates (n = 29, including the 6 isolates from genitourinary sites), but 27/29 (93.1%) isolates had insufficient data from experimental studies to estimate the coverage of the MenB vaccine Bexsero (Appendix 2 Table 2). We predicted cross-reactivity to the MenB vaccine Trumenba for 18/29 (62.1%) isolates. For the MenB disease isolates from China, 7/17 (41.2%) were deemed cross-reactive with Trumenba; however, we had insufficient data for the remaining 10/17 (58.8%) to determine reactivity.

### Molecular Markers of Strains from Europe Infecting MSM

None of the CC4821 isolates harbored frameshifted *fHbp* allele or *penA327*, but the distribution of putatively functional AniA proteins was diverse. The *aniA* gene was absent in all L44.1 isolates (Figure 3) but was present in all of the other 123 CC4821 isolates, of which 96.7% (119/123) isolates harbored putatively functional AniA proteins (Figure 5; Appendix 2 Figures 16–17).

### Evolution of Sublineage L44.1 (China^CC4821-R1-C/B^ Clone)

Five specific loci were present in >90% of L44.1 but in <10% of other sublineages. These loci were involved in signaling and cellular processes (n = 2), metabolism (n = 1), and genetic information processing (n = 1) (Table 3). No loci were specific to any of other 3 sublineages.

**Table 3:** Loci present only in sublineage L44.1 of clonal complex 4821 of Neisseria meningitidis*

Locus	Gene ID	Product	KEGG pathway
NEIS0364	NMCC_0368	Conserved hypothetical protein	Genetic information processing
NEIS0365	NMCC_0369	Competence protein ComFC	Signaling and cellular processes
NEIS0632	NMCC_0638	Lipoprotein	Signaling and cellular processes
NEIS2493	NMBG2136_1300	Hypothetical integral membrane protein	Not included
NEIS3165	NMCC_2037	Histone macro-H2A1-related protein	Metabolism

*KEGG, Kyoto Encyclopedia of Genes and Genomes.

Prediction of HGT events contributing to the emergence of L44.1 using Gubbins discovered 126 events involving 686 loci shared by the 50 L44.1 isolates (Appendix 2 Figure 18). These events included 216 loci with alleles specific to L44.1. We discovered an additional 83 unique loci based on analysis of the accessory genome. Therefore, a total of 299 unique loci were identified in L44.1; of those, 139 (46.5%) were involved in metabolic function (Appendix 1 Table 3). These 299 unique loci were distributed across the chromosome; we observed 44 areas (216 loci) harboring contiguous loci with unique alleles (Figure 7), among which the exact donors of 36 areas across 149 loci were identified in 46 putative HGT events. The total length of these putative recombination fragments was ≈225 kb, including 87 kb (38.7%) originating from the C:ST-9514 cluster isolates in China during 1966–1977, followed by 25 kb (11.1%) from MenA isolates (CC5 and CC1) in China during 1966–1984 (Table 4).

**Table 4:** Potential donors of horizontal gene transfer events associated with contiguous unique loci in sublineage 44.1 of clonal complex 4821 of Neisseria meningitidis*

Event	Donor strain (serogroup: ST)	Clonal complex	Country (year)	PubMLST ID	Fragment size, bp	Position†	NEIS no. unique locus
1	Nm281 (W: ST9486)	CC174	China (1977)	19343	2,369	1–2369	NEIS2139, NEIS2140, NEIS2141
2	Nm284 (E: ST9500)	Singleton	China (1977)	19394	5,188	20775–25962	NEIS0007, NEIS0008
3	NmR29026 (C: ST4822)	ST9514 cluster	China (1966)	86023	3,242	27345–30586	NEIS0009
4	Nm420815 (X: ST5586)	Singleton	China (2008)	85974	5,329	114259–119587	NEIS2052, NEIS2050, NEIS2049
5	Nm252 (B: ST1790)	CC41/44	China (1976)	26654	9,627	210681–220307	NEIS1972, NEIS1968, NEIS1967, NEIS1966
6	Nm284 (E: ST9500)	Singleton	China (1977)	19394	3,886	244692–248577	NEIS1956, NEIS2479, NEIS1954, NEIS1952
7	NmR29026 (C: ST4822)	ST9514 cluster	China (1966)	86023	2,180	282425–284604	NEIS1916, NEIS1915, NEIS1914
8	BZ 232 (B: ST38)	CC37	The Netherlands (1964)	410	4,349	313176–317524	NEIS0313, NEIS0314
9	Nm289 (C: ST4822)	ST9514 cluster	China (1977)	19721	5,828	321318–327145	NEIS0320, NEIS0321, NEIS0323
10	Nm259 (B: ST17)	Singleton	China (1977)	26658	5,885	341967–347851	NEIS0340, NEIS0341, NEIS0342
11	NmR29026 (C: ST4822)	ST9514 cluster	China (1966)	86023	1,371	360040–361410	NEIS0355, NEIS0356
12	NmNX21 (B: ST5655)	CC8	China (1987)	86022	5,346	365048–370393	NEIS0363
13	NmHE89009 (B: ST5642)	Singleton	China (1989)	86012	3,549	372802–376350	NEIS0367, NEIS0370
14	NmHE89009 (B: ST5642)	Singleton	China (1989)	86012	1,076	383868–384943	NEIS0375
15	Nm218 (B: ST10385)	Singleton	China (1976)	26633	3,652	386208–389859	NEIS0378, NEIS0379
16	W10470 (Y: ST4545)	Singleton	South Africa (2017)	59858	3,819	410887–414705	NEIS0394, NEIS0395, NEIS0396
17	Nm148 (B: ST32)	CC32	China (1967)	26613	1,532	479638–481169	NEIS0458, NEIS0459
18	200067 (B: ST3088)	CC35	Bangladesh (2009)	94107	1,602	481200–482801	NEIS0462
19	Nm279 (C: ST9506)	ST9514 cluster	China (1977)	19400	7,850	573877–581726	NEIS0548, NEIS0550, NEIS0551, NEIS0553, NEIS0555
20	NmR29026 (C: ST4822)	ST9514 cluster	China (1966)	86023	3,860	595670–599529	NEIS0567, NEIS0569, NEIS0570
21	P15 (B: ST32)	CC32	Norway (1969)	26056	8,751	609318–618068	NEIS0581, NEIS0582, NEIS0583, NEIS0586
22	Nm441102 (B: ST8929)	Singleton	China (2010)	85986	10,176	632301–642476	NEIS0612, NEIS3022, NEIS0613, NEIS0615, NEIS0617, NEIS0618, NEIS0619, NEIS0620
23	9281 (C: ST8447)	CC175	South Africa (2003)	40583	8,269	657348–665616	NEIS0641, NEIS0643, NEIS0644, NEIS0645, NEIS0649
24	Nm279 (C: ST9506)	ST9514 cluster	China (1977)	19400	6,236	704873–711108	NEIS0685, NEIS0686, NEIS0689, NEIS0690
25	NmR29026 (C: ST4822)	ST9514 cluster	China (1966)	86023	4,663	725942–730604	NEIS0711, NEIS0713, NEIS0714
26	11–004 (A: ST5)	CC5	China (1984)	82	5,393	739214–744606	NEIS0723, NEIS0724, NEIS0729, NEIS0730
27	61106 (A: ST1)	CC1	Niger (1961)	34552	8,069	739627–747695	NEIS0731, NEIS0733
28	NmR29026 (C: ST4822)	ST9514 cluster	China (1966)	86023	2,412	1038607–1041018	NEIS1076, NEIS1078
29	NmR29026 (C: ST4822)	ST9514 cluster	China (1966)	86023	16,089	1091594–1107682	NEIS1127, NEIS1128, NEIS1129, NEIS1131, NEIS3081, NEIS1132, NEIS1133, NEIS1134, NEIS1137, NEIS1138, NEIS1139
30	NmR29026 (C: ST4822)	ST9514 cluster	China (1966)	86023	10,667	1130089–1140755	NEIS1160, NEIS1165, NEIS1168
31	M10 240614 (B: ST9817)	singleton	UK (2010)	20020	2,533	1198534–1201066	NEIS1229, NEIS1231, NEIS1232, NEIS1233
32	C10695 (cnl: new ST)	CC1136	South Africa (2017)	59854	1,852	1491092–1492943	NEIS1479, NEIS1480
33	Nm279 (C: ST9506)	ST9514 cluster	China (1977)	19400	4,194	1585327–1589520	NEIS1568, NEIS1570, NEIS1571
34	NmR29026 (C: ST4822)	ST9514 cluster	China (1966)	86023	4,306	1611638–1615943	NEIS1590, NEIS3123, NEIS1594
35	NM320 (B: ST9513)	Singleton	China (1977)	19407	1,618	1630175–1631792	NEIS1605
36	NmGZ80028 (A: ST3)	CC1	China (1974)	86004	9,784	1644769–1654552	NEIS1618, NEIS1619, NEIS1622, NEIS1624, NEIS1629, NEIS1630
37	NmR29026 (C: ST4822)	ST9514 cluster	China (1966)	86023	1,537	1674337–1675873	NEIS1647
38	8044 (C: new ST)	Singleton	South Africa (2002)	40541	3,081	1675114–1678194	NEIS1649, NEIS1650
39	Nm279 (C: ST9506)	ST9514 cluster	China (1977)	19400	3,799	1840253–1844051	NEIS1819, NEIS1820, NEIS1822, NEIS1824
40	Nm140 (B: S-351)	Singleton	China (1967)	26610	2,696	1926888–1929583	NEIS0264, NEIS0261, NEIS0260
41	6183 (C: ST13352)	Singleton	South Africa (2001)	40534	3,884	1930185–1934068	NEIS0257
42	NmGZ80028 (A: ST3)	CC1	China (1974)	86004	2,392	2076119–2078510	NEIS0108, NEIS0107
43	Nm280 (C: ST9514)	ST9514 cluster	Chna (1977)	52352	6,321	2079333–2085653	NEIS0105, NEIS0104, NEIS3165, NEIS0102, NEIS0101, NEIS0100
44	Nm140 (B: ST351)	Singleton	China (1967)	26610	6,681	2094786–2101466	NEIS2071, NEIS2074, NEIS2075, NEIS2078, NEIS2079
45	NmR29026 (C: ST4822)	ST9514 cluster	China (1966)	86023	2,610	2101520–2104129	NEIS2080, NEIS2081, NEIS2082
46	80179 (E: ST178)	CC178	France (1980)	34575	6,049	2117485–2123533	NEIS2106, NEIS2107, NEIS2108, NEIS2109, NEIS2110, NEIS2112

*CC, clonal cluster; ST, sequence type,  †Positions are given according to the genome sequence of *N. meningitidis* strain 053442 (GenBank accession no. NC_010120.1).

### Evolution of CC4821 Isolates from Outside China

We identified 49 CC4821 isolates from countries outside of China, and most (40/49, 81.6%) were assigned to L44.3, of which there were 39 MenB and 1 MenC, constituting the distinct Europe–USA cluster (Figures 2, 5). The representative molecular characteristics of the Europe–USA cluster was B:P1.17-6,23: F3-36:ST-3200(CC4821); its antigen gene profile was *porA*-423, *fHbp*-16, *nhba*-553, *porB*-265, *fetA*-1069, *opcA*-100, *nspA*-26, *tbpA*-1333, and *NMB0315*-194 and antimicrobial resistance profile *gyrA*-12, *parC*-275, *penA*-9 with PBP2 mutations, *ponA*-7, and *rpoB*-85 (Figure 6). In Gubbins analysis, 33 events involving 193 loci were shared by all the Europe–USA cluster isolates (Appendix 2 Figure 18); we discovered 60 unique loci for which we could not identify their potential donors. These unique loci were involved in functions mainly associated with metabolism (23/60, 38.3%) and genetic information processing (18/60, 30%) (Appendix 1 Table 4).

In addition to the 40 Europe–USA cluster isolates, there were 6 MenC invasive isolates from India (n = 4, identified 2014–2016), Japan (n = 1, identified in 2017), and New Zealand (n = 1, identified in 2018). These 6 isolates were clustered together and were closely related with 44 isolates from China within sublineage L44.1 (Figures 2–3). Only the isolate from Japan showed the typical molecular feature of Anhui outbreak strain (C:P1.7-2,14:F3-3:ST-4821[CC4821]).

### Features and Evolution of Serogroup W CC4821 Isolates

A total of 11 MenW isolates from China were identified; the representative strain designation was W:P1.5-3,10-2:F1-7:ST-8491(CC4821), with similar gene profiles of antigen-encoding loci (*porA*-1804, *fHbp*-474, *nhba*-966, *fetA*-37, *opcA*-4, *nspA*-117, and *NMB0315*-21) and antimicrobial resistance loci (*gyrA*-294 with T91I, *parC*-779, *ponA*-7, and *rpoB*-85). These MenW isolates constituted a distinct cluster in L44.4; they were more closely related to NM193 (C:P1.20-3,23-1:F1-5:ST-3436[CC4821], dating from 1972) than to NM205 (C:P1.20,23-2:F5-135:ST-4821[CC4821], dating from 1973) (Appendix 2 Figure 17).

## Discussion

The meningococci can cause IMD, leading to endemic disease in most if not all human populations. Several genotypes belonging to hyperinvasive lineages, in combination with the disease-associated capsular serogroups, can cause elevated levels of disease; some of which also possess epidemic and pandemic potential. In the past 100 years, notable epidemics and pandemics have included meningococci such as A:CC1, A:CC5, B:CC41/44, C:CC11, and W:CC11 (*35*). Here, we employed a genomic analysis of MenB, MenC, and MenW CC4821 isolates dating from 1972–2019 to assess their epidemic and pandemic potential. Of special concern are the expansion of the quinolone-resistant clone China^CC4821-R1-C/B^ from China to other countries; the potential possession of universal resistance to penicillin in Europe–USA cluster isolates; and the uncertainty over the potential efficacy of existing vaccines to prevent B:CC4821 diseases.

CC4821, which corresponds to lineage 44, shares several properties in common with the hyperinvasive CC11 meningococci (Lineage 11): its ability to express several serogroups, global distribution, colonization of urogenital and anorectal tracts, and separation into distinct sublineages. CC11 has caused well-documented epidemics and pandemics on several occasions, including US military outbreaks in the 1960s; Hajj-associated outbreaks in 2000s; and the global epidemics from 2010, especially outbreaks among MSM (*34*–*38*). These similar characteristics raise the concern that the CC4821 may have the potential to cause similar global pandemics.

Consistent with the presence of the epidemic CC4821 clone in countries outside of China, 6 CC4821 IMD meningococci from India, Japan, and New Zealand, isolated during 2014–2018, clustered with China^CC4821-R1-C/B^ meningococci in L44.1 (Figure 3). IMD cases caused by these 6 isolates were all found in native inhabitants (*10*,*15*,*39*); all 6 isolates shared similar serogroup, antibiotic, and antigen (except *porA* and *fHbp*) gene characteristics with isolates from China in this sublineage. In particular, all 6 isolates harbored the T91I mutation in GyrA, the molecular marker of quinolone resistance, compatible with their quinolone-resistant phenotype (*10*,*15*,*39*). These isolates also had strain-specific features, which suggested that they resulted from transmission from the China^CC4821-R1-C/B^ clone. The isolate from Japan, which had the typical molecular features of the Anhui outbreak strain (C:P1.7-2,14:F3-3:ST-4821[CC4821]) (*4*), became the earliest-reported quinolone-resistant meningococcus harboring ParC mutation (S87I, allele 1538) to cause IMD worldwide (*10*). In contrast, none of the China^CC4821-R1-C/B^ clone isolates from China had ParC mutations conferring antimicrobial resistance. The isolate from Japan was more closely related to the reference strain 053442 than were the 4 India and 1 New Zealand isolates, which had different STs, *porA*, *fHbp,* and *tbpA* alleles (Figure 4). 

Although we did not identify a putative ancestor of the quinolone-resistant clone China^CC4821-R1-C/B^ in this study, we found 299 loci with alleles unique to this sublineage. Approximately half of these loci were associated with metabolic pathways, suggesting that divergence in metabolic genes may play a role in the emergence of epidemic meningococci. Several studies have indicated that metabolic genes can influence the pathogenesis and virulence of the meningococcus, for example by allowing alternative host resources to be exploited in invasive disseminated infections (*40*). Changes in the hyperinvasive A:CC5 meningococci circulating in Africa have been associated with HGT of core genes involved in metabolic processes (*41*). The putative donors of these unique alleles included lineages from different serogroups and dates of isolation, such as C:ST-9514 cluster, 1960s–1970s; A:CC5 and A:CC1, 1960s–1980s; B:CC32, 1960s; B:CC41/44, 1970s; and E:CC178, 1980s (Table 4). The C:ST-9514 cluster, STs that do not presently form part of a clonal complex documented in PubMLST, has ST9514 as the central ST and was predominant in MenC carriage isolates during 1965–1980 in Shanghai, China (*42*). Therefore, the emergence of China^CC4821-R1-C/B^ clone was perhaps associated with accumulation of these unique alleles, which accounted for the separation from other sublineages in the allele-based phylogeny (Figure 2).

In the PubMLST database, >60% of the CC4821 isolates from outside China were MenB. Of these, 49 genomes were available in this study, including isolates from IMD (n = 15) and urogenital and rectal tracts (n = 6). Most of these genomes clustered in sublineage L44.3 and constituted a distinct cluster, the Europe–USA cluster, showing the typical strain designation: B:P1.17-6,23-x:F3-36:ST-3200(CC4821), wherein 23-x refers to 23, 23-2, and 23-6. The PorA and FetA types P1.17-6,23-x and F3-36 were only found in this cluster. The *Neisseria* PubMLST database had no genome data for 24 CC4821 isolates from other countries (United States, Brazil, France, Czech Republic, Spain, Italy, Australia, and Vietnam), but included PorA or FetA variants for the 24 isolates (Appendix 1 Table 5). Of these, 19 (79.2%) exhibited P1.17-6,23-x or F3-36, suggesting they might belong to the Europe–USA cluster. This cluster was distinct from the epidemic clone China^CC4821-R1-C/B^. For example, the antigen profile characteristic of the Europe–USA cluster was P1.17-6,23-x, F3-36, PorB-3-229, *fHbp*-16, *nhba*-553, *opcA*-100, *nspA*-26, and *tbpA*-1333, compared with P1.7-2,14, F3-3, PorB-3-48, *fHbp*-498, 22 and 489, *nhba*-124, *opcA*-4, *nspA*-4, and *tbpA*-7 in the China^CC4821-R1-C/B^ clone. In addition, all the China^CC4821-R1-C/B^ isolates harbored the mutation T91I in GyrA, whereas almost all of the Europe–USA cluster isolates possessed mutations in PBP2 (F504L, A510V, I515V, H541N, and I566V). This may reflect different antibiotic selective pressures experienced by the Europe–USA and the China^CC4821-R1-C/B^ meningococci. Penicillins were the most-used antimicrobial drugs in outpatients in Europe, whereas China has the second largest global increases of fluoroquinolone consumption (*43*,*44*). A high frequency (>70%) of quinolone resistance has been reported in China since 2005 (*5*), whereas 65% of meningococci in Europe showed reduced susceptibility to penicillin G during 1945–2006 (*45*). In the 2 oldest isolates of the sublineage L44.3, Nm282 (B:P1.20,23:F3-36:ST-3200[CC4812]) was much closer to the Europe–USA cluster isolates than Nm323 (B:P1.20,23:F3-36:ST-5798[CC4821]) (Figure 5), and it seemed more likely to be the ancestor of the Europe–USA cluster isolates.

Urogenital and rectal meningococci have raised increasing public health concerns (*34*). In 2017, CC4821 anorectal isolates were identified in the United Kingdom (*12*). In this study, we identified CC4821 isolates from urethral and rectal tracts that clustered with isolates from IMD specimens and oropharyngeal carriage (Figure 5). With the exception of L44.1 isolates, most of the CC4821 isolates contained a putatively functional nitrite reductase (AniA), required for growth in anaerobic environments. The CC4821 isolates acquired quinolone resistance alleles from *N. lactamica* and *N. subflava* (*46*); the ability to grow in anaerobic environments will facilitate acquisition of gonococcal alleles, including antimicrobial resistance alleles. Such events seem to have already occurred in a sublineage of CC11, which was responsible for several IMD outbreaks and urethritis among MSM (*34*). They shared the same *penA* allele (*penA327/penAXXXIV*) with gonococcal bacteria and showed decreased susceptibility to third-generation cephalosporins (*47*). Although PubMLST is the largest global repository of meningococcal genomes (>22,000), a paucity of genomic data were available from isolates originating from the genitourinary or respiratory tract, suggesting an underestimation of the global dissemination of CC4821. Therefore, we recommend WGS for urogenital-, rectal-, and respiratory-derived meningococci if they are exhibiting antimicrobial resistance.

CC4821 lineage 44 includes isolates from different serogroups, including MenB, MenC, and MenW. In China, MenC and MenW isolates can be prevented by vaccines, such as group A and C meningococcal polysaccharide vaccine (MPV-AC) and MPV-ACYW, but no routinely administered vaccine is available to prevent MenB IMD (*48*). Two protein-based vaccines targeting MenB meningococci, 4CMenB (Bexsero) and rLP2086 (Trumenba), have been licensed in several countries (*49*–*50*; reference *51 *in Appendix 2), but limited data are available on the bacterial coverage of these vaccines to CC4821 isolates directly from serum bactericidal activity assays, the Meningococcal Antigen Typing System (MATS) for Bexsero, or meningococcal antigen surface expression for Trumenba. One B:CC4821 isolate (M14-240580, UK) was reported to be tested using the MATS assay and showed no potential protection (reference 52 in Appendix 2). Using systems to index complex genotypic and phenotypic data, such as the MenDeVAR Index, we predicted that ≈60% of B:CC4821 disease-causing isolates might be prevented through vaccination with Trumenba; data are insufficient to infer Bexsero reactivity. Further testing of globally diverse meningococci is needed with these experimental assays to analyze potential vaccine impact in settings outside Europe.

In summary, we have undertaken a comprehensive genomic analysis of a hyperinvasive meningococcal CC4821 expressing MenB, MenC, and MenW with expansion from China to other global geographic locations with currently available genomic data. We identified key genomic factors and putative evolutionary changes that might have led to the emergence and persistence of the epidemic quinolone-resistant clone in China. Vaccine coverage for MenB CC4821 isolates needs further evaluation. Enhanced laboratory surveillance for CC4821 isolates from IMD cases and from oropharyngeal, urethral, and rectal carriage is needed to monitor global trends of expansion, which will be essential for local immunization policies.

Appendix 1 Additional data for about ST4821 clonal complex hyperinvasive, quinolone-resistant meningococcus bacteria.

Appendix 2 Additional information about ST4821 clonal complex hyperinvasive, quinolone-resistant meningococcus bacteria.
