# Supplementary material for: Evolution of Sequence Type 4821 Clonal Complex Hyperinvasive and Quinolone-Resistant Meningococci
Source: Emerg Infect Dis. 2021 Apr;27(4):1110–22. doi: 10.3201/eid2704.203612 (PMC8007298; doi:10.3201/eid2704.203612)
Supplement: Appendix 2 — Additional information about ST4821 clonal complex hyperinvasive, quinolone-resistant meningococcus bacteria. [file 20-3612-Techapp-s2.pdf]

Article DOI: https://doi.org/10.3201/eid2704.203612

Evolution of Sequence Type 4821 Clonal Complex Hyperinvasive and Quinolone-Resistant Meningococci

Appendix 2

Additional References

51. Findlow J, Nuttens C, Kriz P. Introduction of a second MenB vaccine into Europe—needs and opportunities for public health. Expert Rev Vaccines. 2019;18:225–39. PubMed https://doi.org/10.1080/14760584.2019.1578217

52. Parikh SR, Newbold L, Slater S, Stella M, Moschioni M, Lucidarme J, et al. Meningococcal serogroup B strain coverage of the multicomponent 4CMenB vaccine with corresponding regional distribution and clinical characteristics in England, Wales, and Northern Ireland, 2007–08 and 2014–15: a qualitative and quantitative assessment. Lancet Infect Dis. 2017;17:754–62. PubMed https://doi.org/10.1016/S1473-3099(17)30170-6

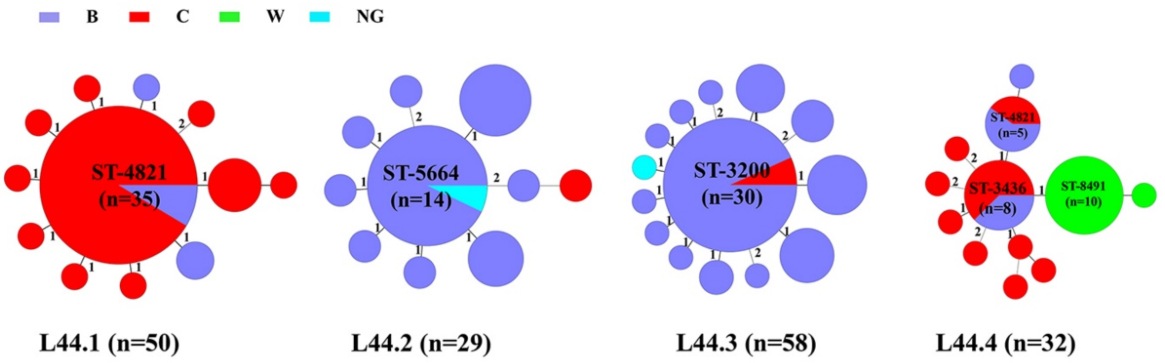

Appendix 2 Figure 1. Central sequence type of each sublineage of *Neisseria meningitidis* ST-4821 clonal complex. The numbers of different loci between the central sequence type (ST) and its neighboring STs are labeled. The ST which differs one locus to the central ST is a single-locus variant (SLV) of the central ST. In sublineage 44.1 (L44.1), ST-4821 (n = 35) and its SLVs (n = 13) account for 96% (48/50); in L44.2, ST-5664 (n = 14) and its SLVs (n = 12) take up for 89.7% (26/29); in L44.3, the percentage of ST-3200 (n = 30) and its SLVs (n = 23) is 91.4% (53/58); in L44.4, the percentage of ST-3436 (n = 8) its SLVs (n = 18) is 81.3% (26/32). B, meningococcus belonging to serogroup B; C, meningococcus belonging to serogroup C; W, meningococcus belonging to serogroup W; NG, nongroupable meningococcus bacteria not identified as any of a serogroup.

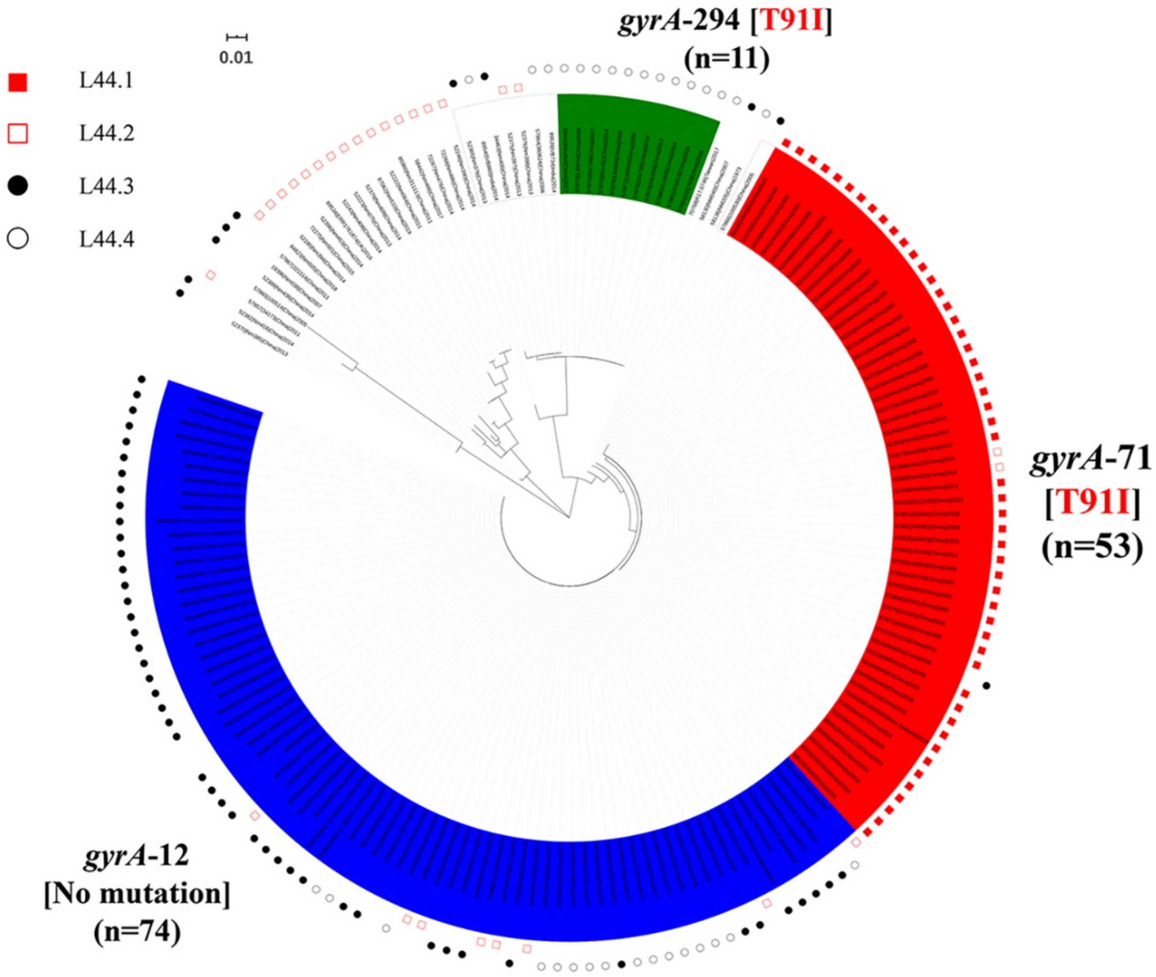

Appendix 2 Figure 2. Phylogenetic analysis of antimicrobial resistance gene sequence variation within the *Neisseria meningitidis* clonal complex 4821 lineage, based on nucleotide sequences of the *gyrA* gene. Scale bar indicates number of base substitutions per site. Blue shading indicates the genomes with *gyrA* allele 12, red shading indicates the genomes with *gyrA* allele 71, green shading, genomes with *gyrA* allele 294.

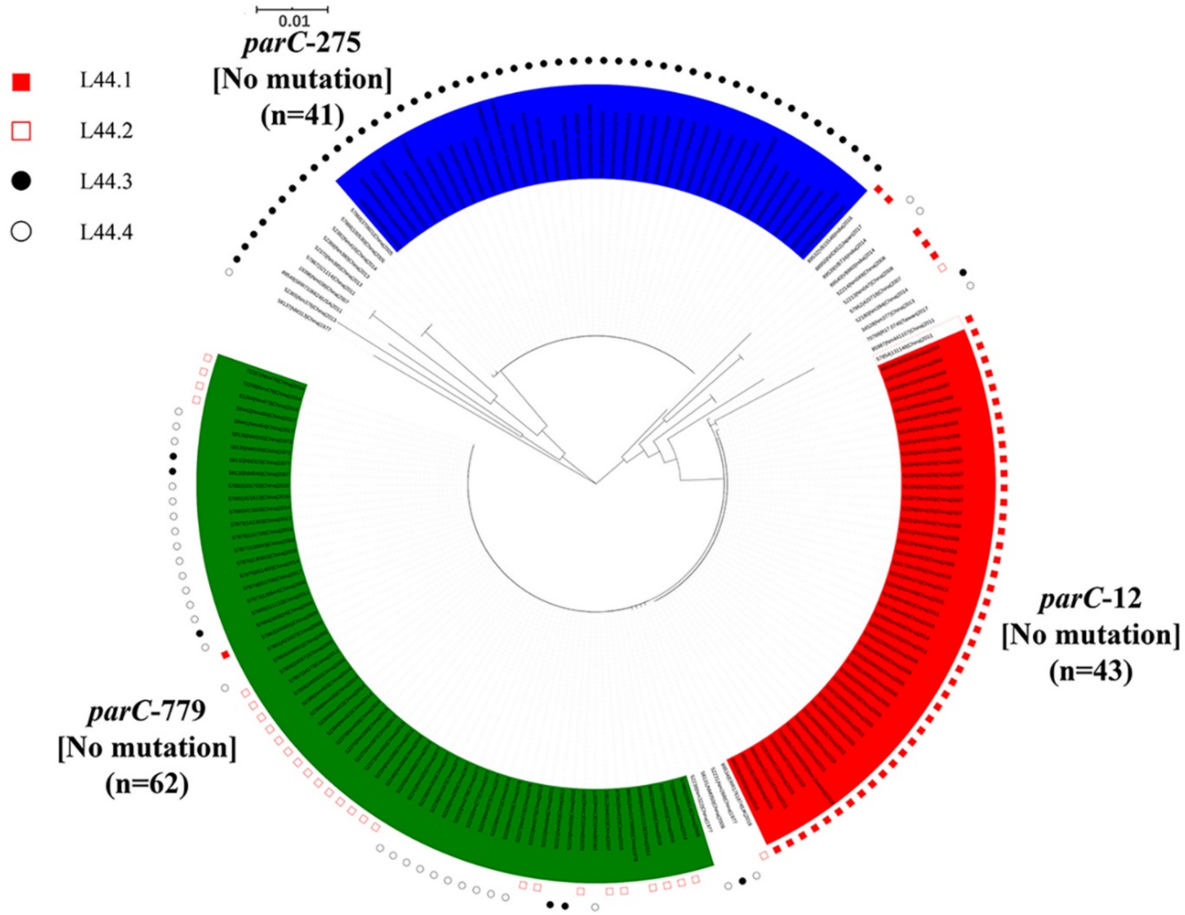

Appendix 2 Figure 3. Phylogenetic analysis of antimicrobial resistance gene sequence variation within the *Neisseria meningitidis* clonal complex 4821 lineage, based on nucleotide sequences of the *parC* gene. Green shading indicates the genomes with *parC* allele 779; red shading indicates genomes with *parC* allele 12; blue shading, genomes with *parC* allele 275.

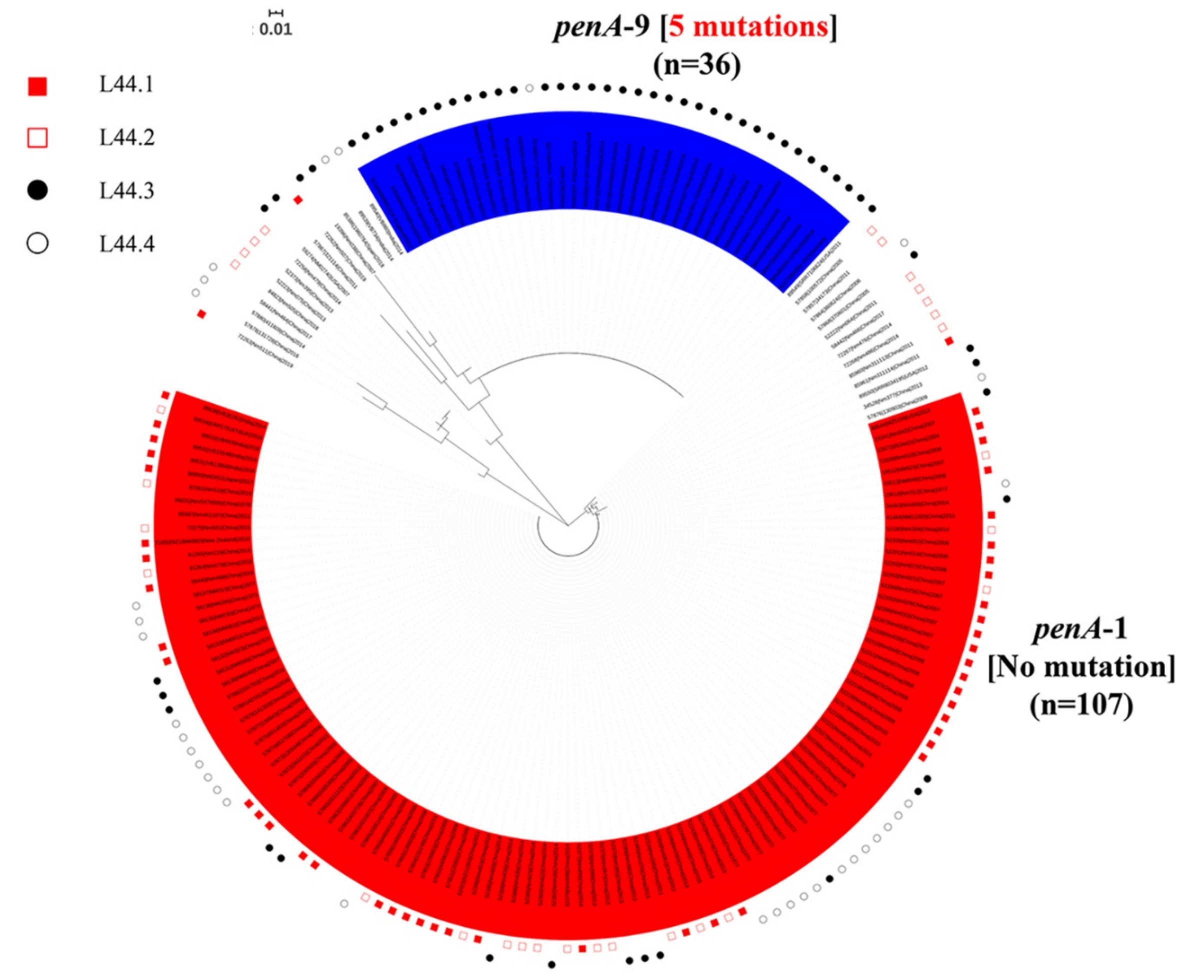

Appendix 2 Figure 4. Phylogenetic analysis of antimicrobial resistance gene sequence variation within the *Neisseria meningitidis* clonal complex 4821 lineage, based on nucleotide sequences of the *penA* gene. The red shading indicates the genomes with *penA* allele 1; blue shading indicates the genomes with *penA* allele 9.

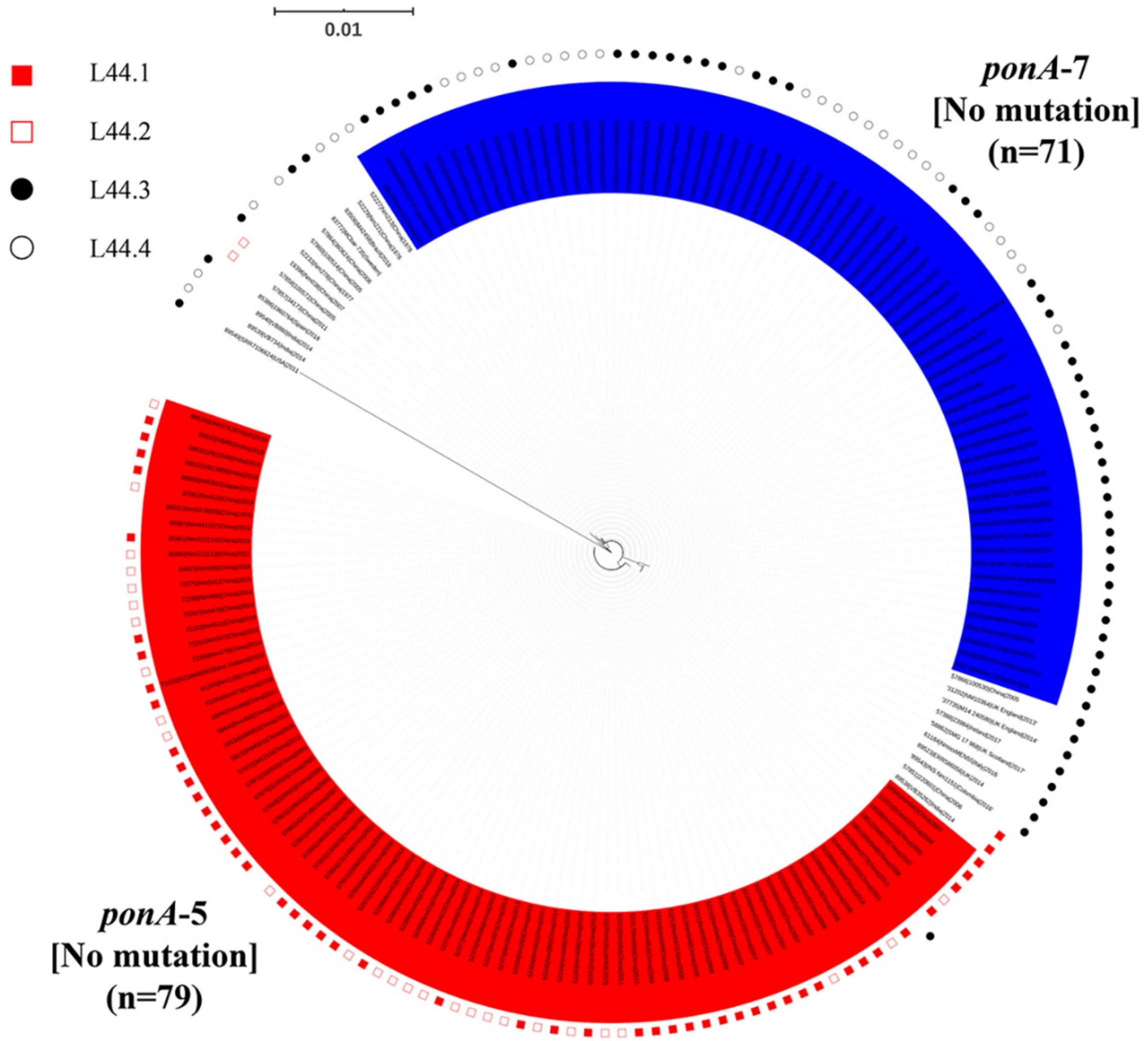

Appendix 2 Figure 5. Phylogenetic analysis of antimicrobial resistance gene sequence variation within the *Neisseria meningitidis* clonal complex 4821 lineage, based on nucleotide sequences of the *ponA* gene. The red shading indicates the genomes with *ponA* allele 5; blue shading indicates the genomes with *ponA* allele 7.

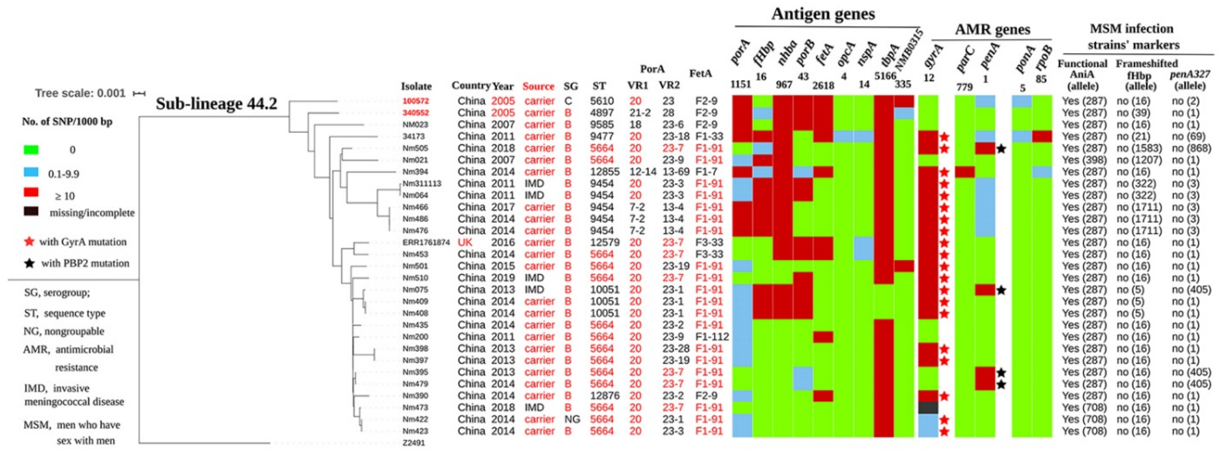

Appendix 2 Figure 6. Phylogenetic analysis of antimicrobial resistance gene sequence variation within the *Neisseria meningitidis* clonal complex 4821 lineage, based on nucleotide sequences of the *rpoB* gene. Gray shading indicates the genomes with *rpoB* allele 85.Scale bar indicates number of substitutions per site.

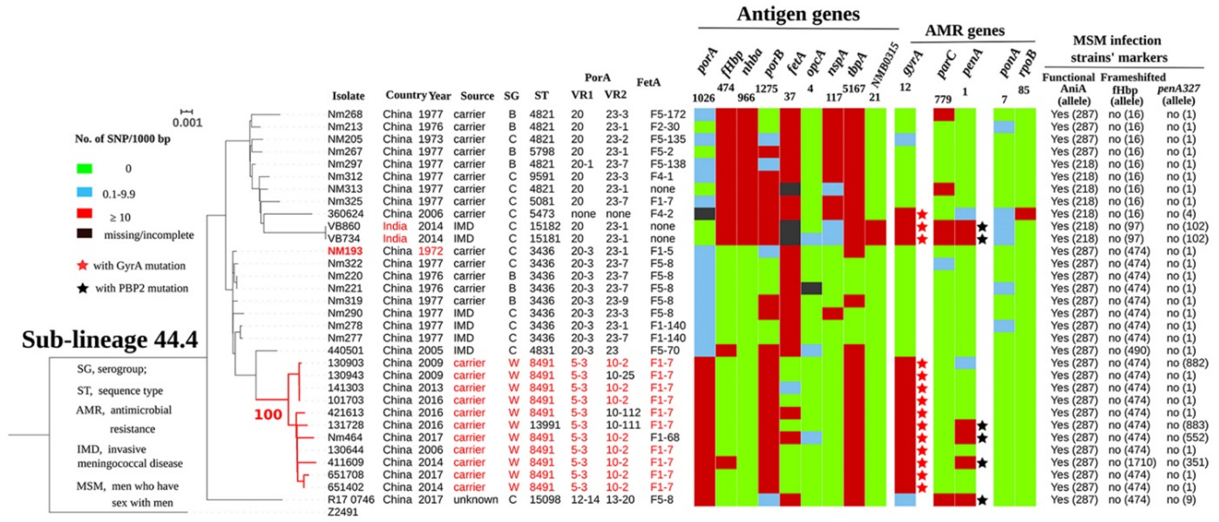

Appendix 2 Figure 7. Phylogenetic analysis of antigen gene sequence variation within the *Neisseria meningitidis* clonal complex 4821 lineage, based on nucleotide sequences of the *porA* gene. A total of 168 *porA* gene sequences were analyzed. Five genome data with incomplete *porA* sequences were excluded, including 57857 (with information of P1.7–2,14 in the PubMLST database), 57864, 57870 (with information of P1.20,23–9 in the PubMLST database), 57871 (with information of P1.7–2,14 in the PubMLST database), and 57872 (with information of P1.7–2,14 in the PubMLST database). 20-x, refers

to 20, 20–1, 20–3. 23-x, refers to 23, 23–1, 23–2, 23–3, 23–6, 23–7, 23–9, 23–19, and 23–28. Gray shading indicates the genomes with PorA subtype P1. 20-x–2, 23-x; blue shading indicates the genomes with P1.17–6, 23-x; red shading with P1.7–2,14; the orange shading with P1.12–1,16-8; yellow shading with P1.5–3,10-2.

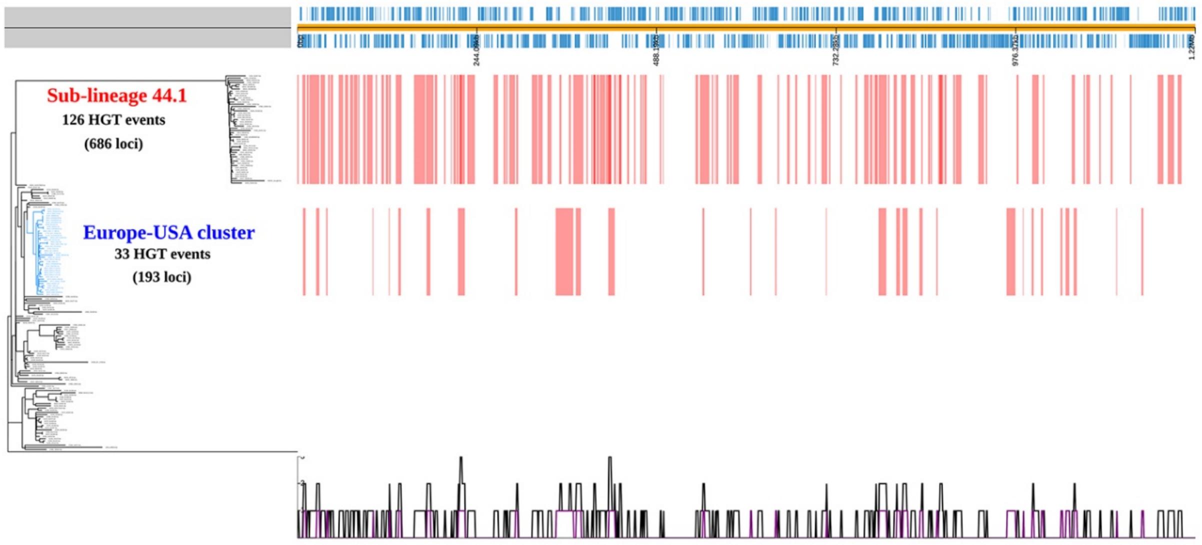

Appendix 2 Figure 8. Phylogenetic analysis of antigen gene sequence variation within the *Neisseria meningitidis* clonal complex 4821 lineage, based on nucleotide sequences of the *fHbp* gene. Gray shading indicates the genomes with *fHbp* allele 16; blue shading indicates the genomes with *fHbp* allele 474;red shading with *fHbp* allele 498; orange shading with *fHbp* allele 489; green shading with *fHbp* allele 22. Scale bar indicates number of base substitutions per site.

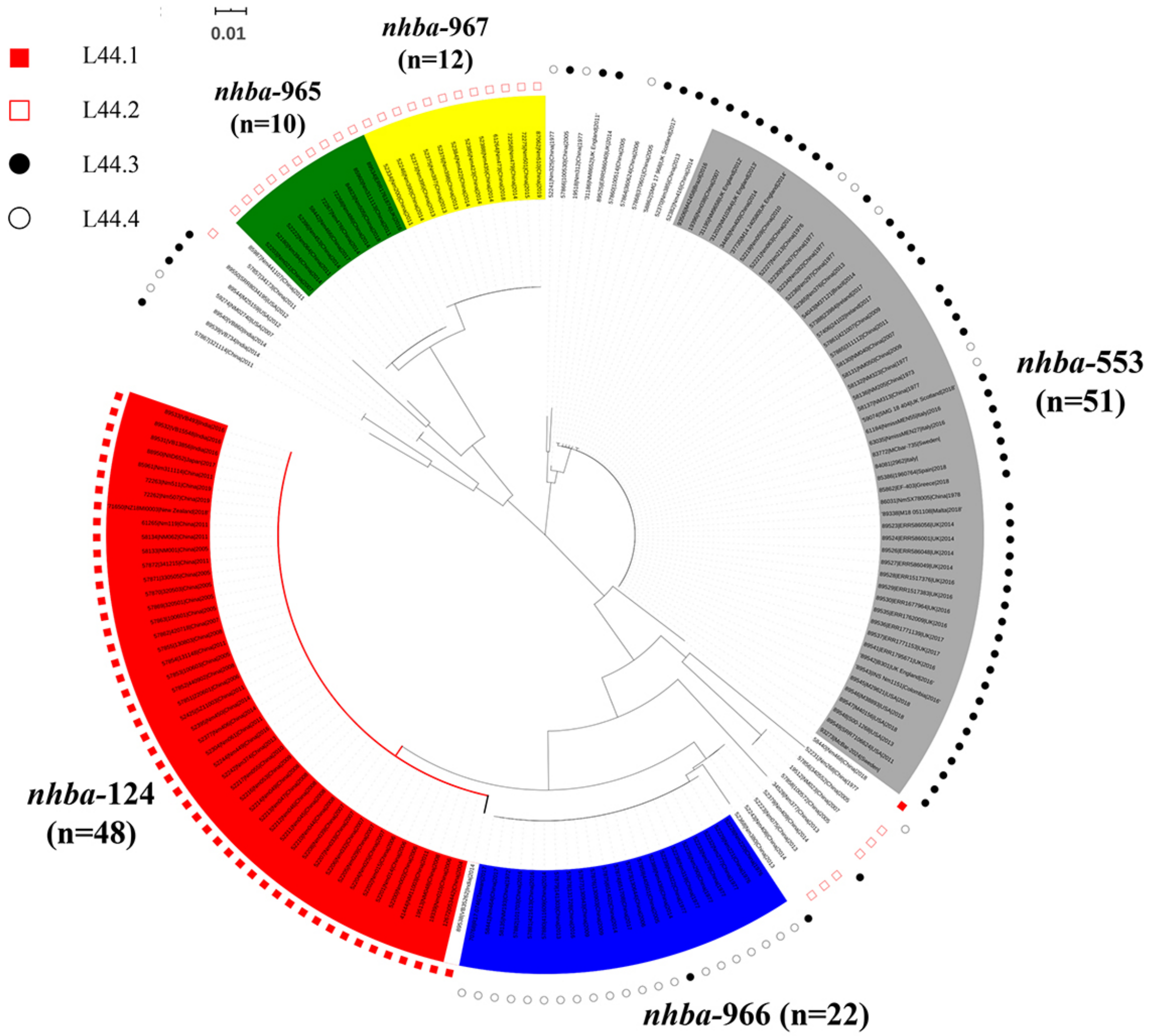

Appendix 2 Figure 9. Phylogenetic analysis of antigen gene sequence variation within the *Neisseria meningitidis* clonal complex 4821 lineage, based on nucleotide sequences of the *nhba* gene. Gray shading indicates the genomes with *nhba* allele 553; red shading with *nhba* allele 124; blue shading indicates the genomes with *nhba* allele 966; orange shading with *nhba* allele 967; green shading with *nhba* allele 965.

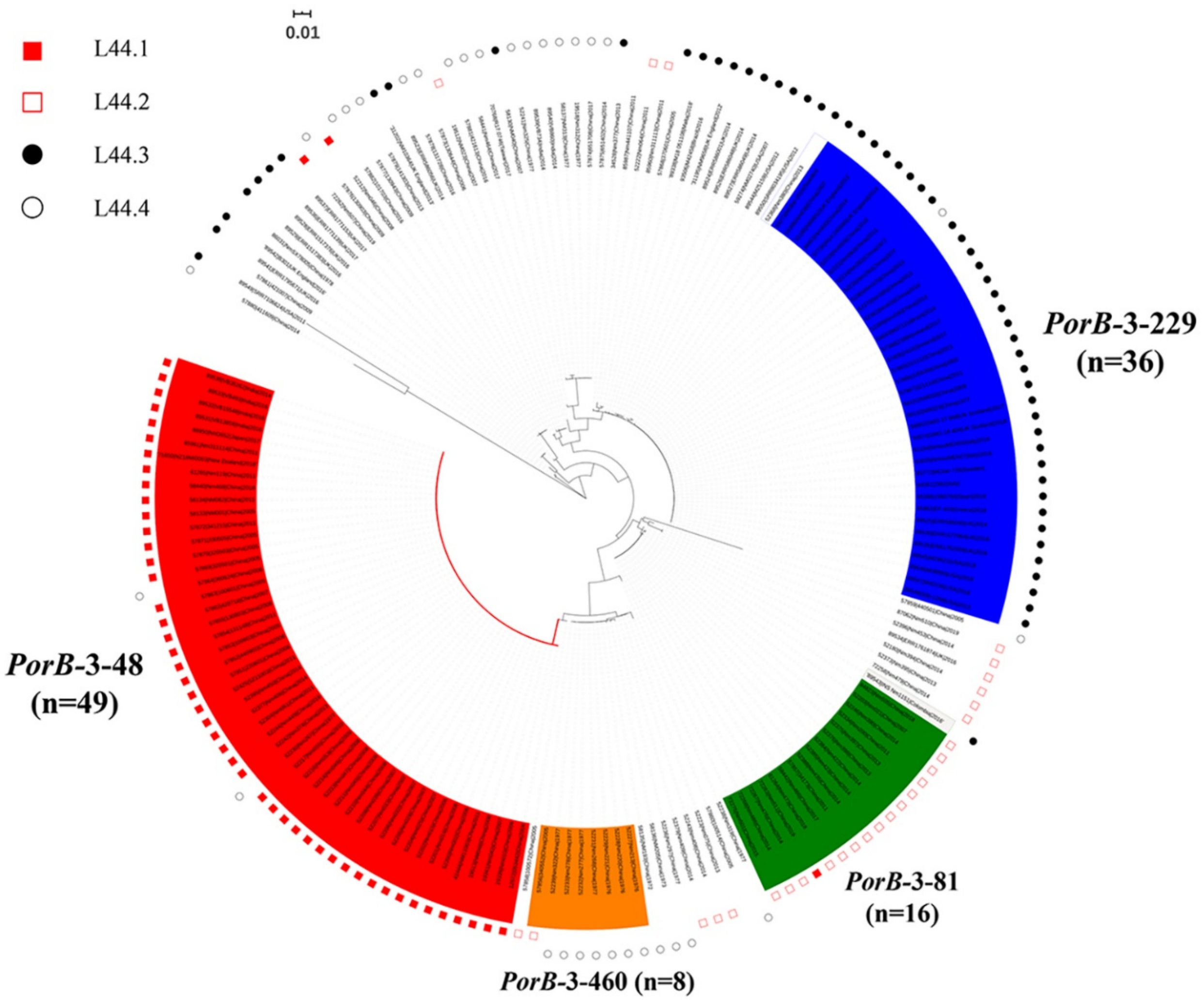

Appendix 2 Figure 10. Phylogenetic analysis of antigen gene sequence variation within the *Neisseria meningitidis* clonal complex 4821 lineage, based on nucleotide sequences of the *porB* gene. Red shading indicates the genomes with PorB peptide 3-48; blue shading indicates the genomes with PorB peptide 3-229; green shading with PorB peptide 3-81; orange shading with PorB peptide 3-460.

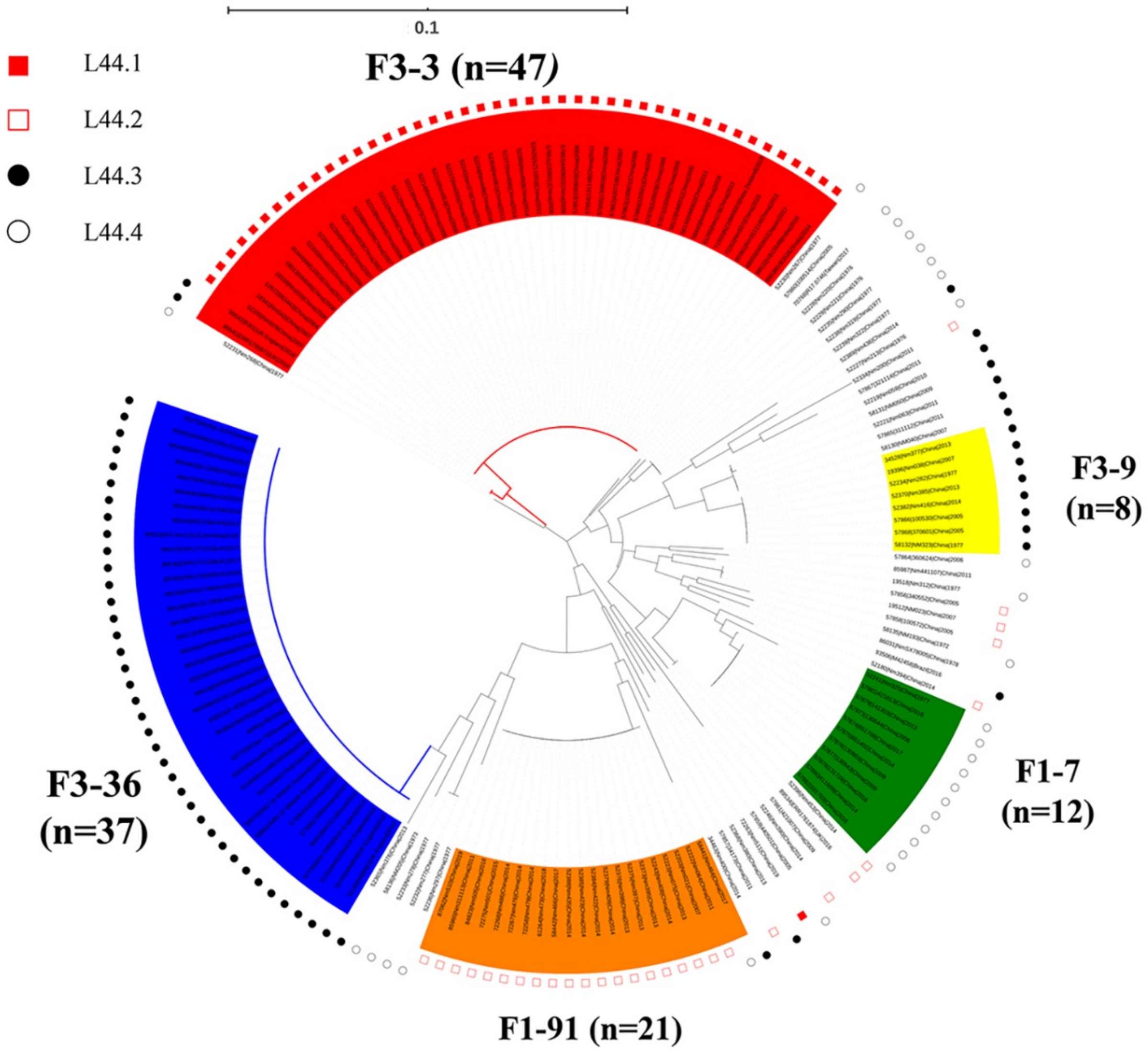

Appendix 2 Figure 11. Phylogenetic analysis of antigen gene sequence variation within the *Neisseria meningitidis* clonal complex 4821 lineage, based on nucleotide sequences of the *fetA* gene. Red shading indicates the genomes with FetA variant 3-3; blue shading indicates the genomes with FetA variant 3-36; the orange shading with FetA variant 1-91; green shading with FetA variant 1-7; yellow shading with FetA variant 3-9.

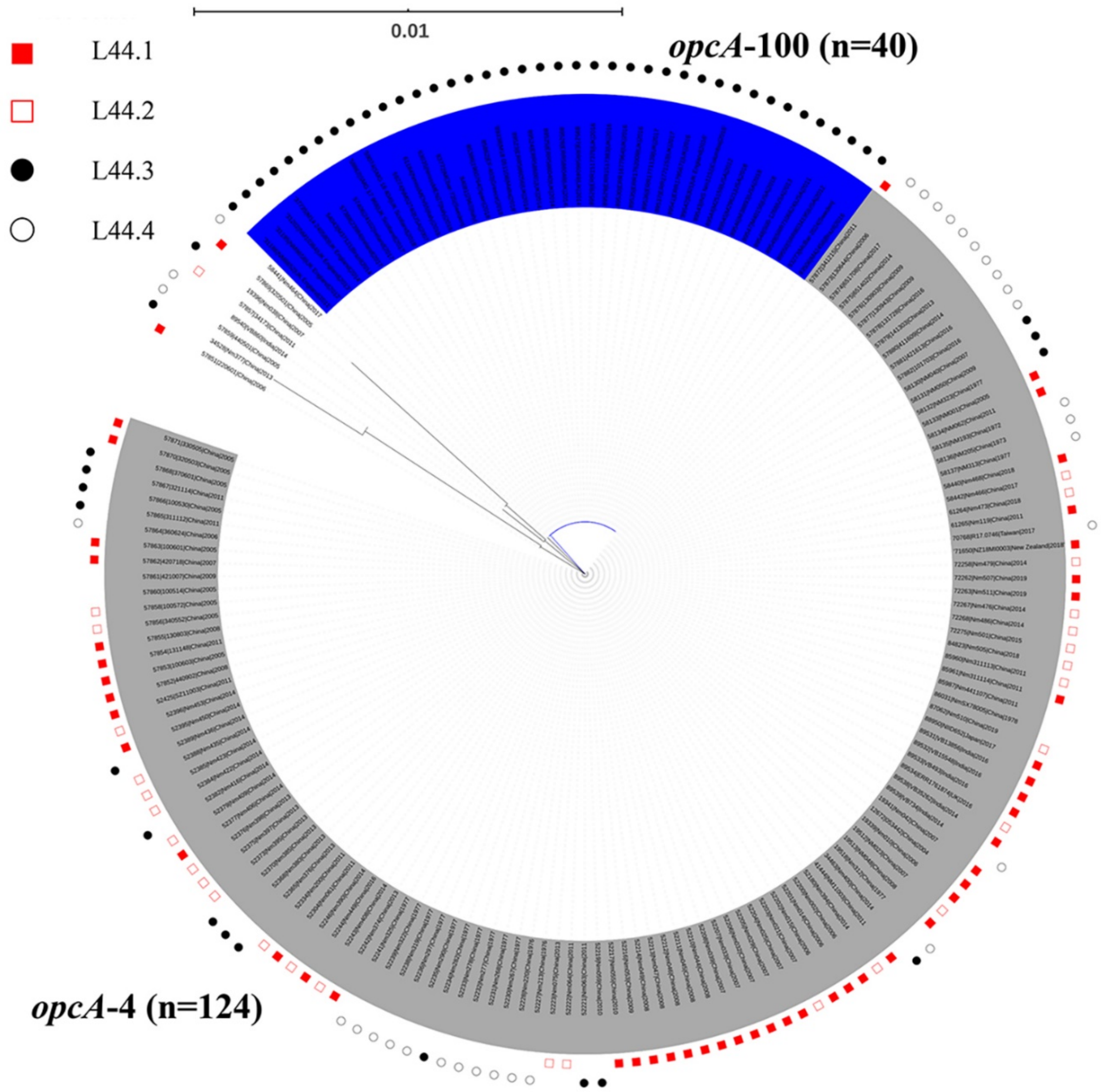

Appendix 2 Figure 12. Phylogenetic analysis of antigen gene sequence variation within the *Neisseria meningitidis* clonal complex 4821 lineage, based on nucleotide sequences of the *opcA* gene. Gray shading indicates the genomes with *opcA* allele 4; blue shading indicates the genomes with *opcA* allele 100.

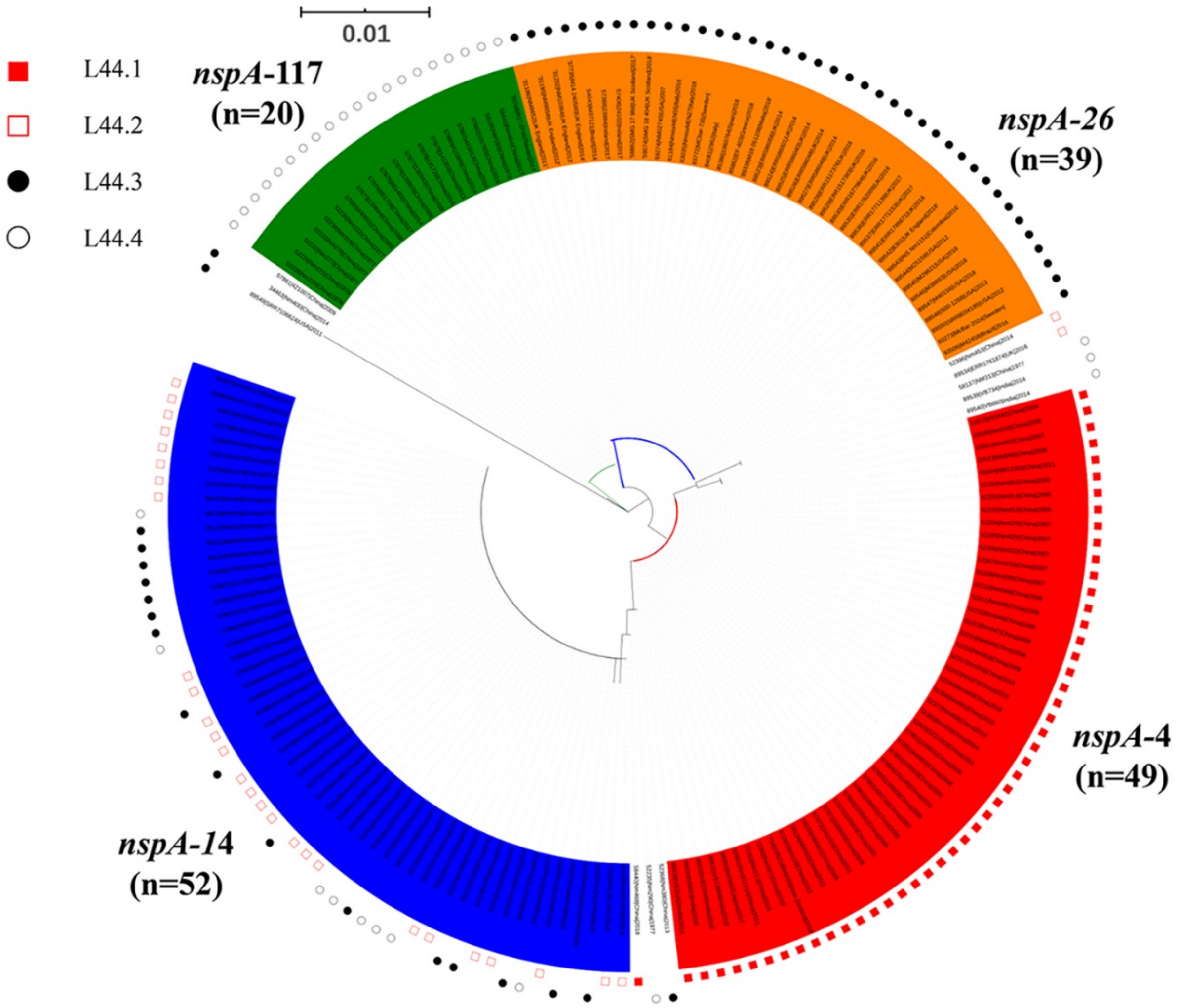

Appendix 2 Figure 13. Phylogenetic analysis of antigen gene sequence variation within the *Neisseria meningitidis* clonal complex 4821 lineage, based on nucleotide sequences of the *nspA* gene. Blue shading indicates the genomes with *nspA* allele 14; red shading with *nspA* allele 4; orange shading with *nspA* allele 26; green shading with *nspA* allele 117.

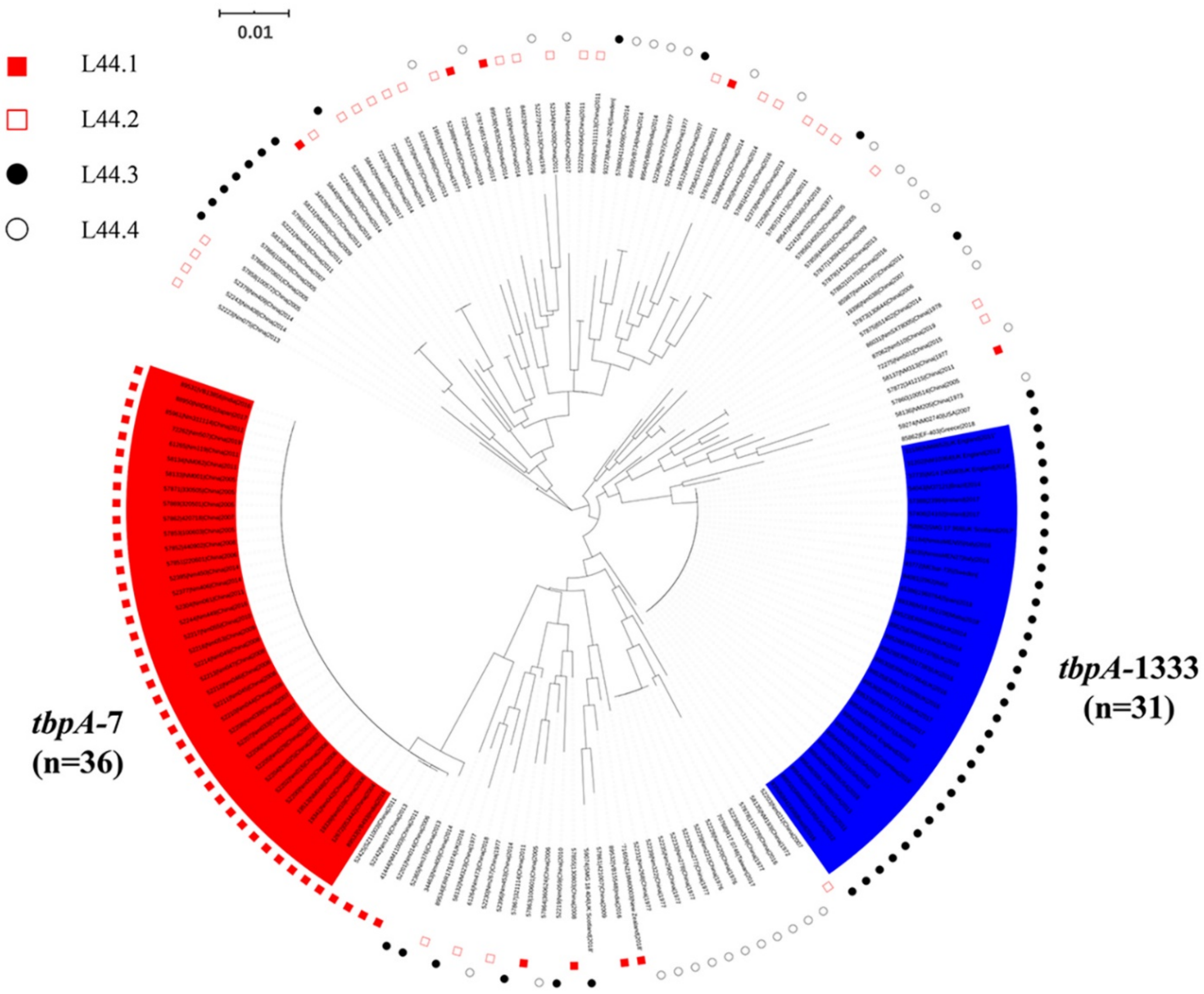

Appendix 2 Figure 14. Phylogenetic analysis of antigen gene sequence variation within the *Neisseria meningitidis* clonal complex 4821 lineage, based on nucleotide sequences of the *tbpA* gene. Red shading indicates the genomes with *tbpA* allele 7; blue shading indicates the genomes with *tbpA* allele 1333.

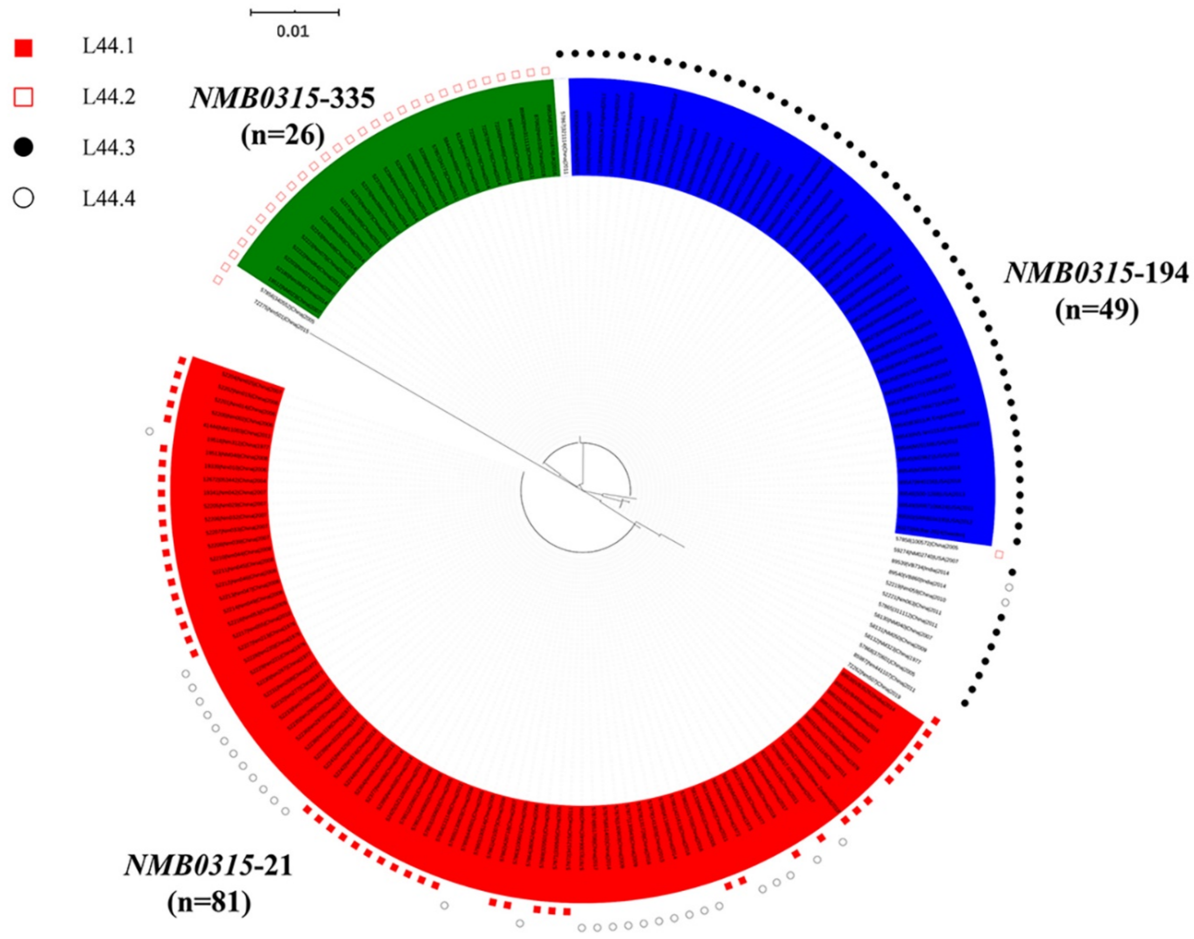

Appendix 2 Figure 15. Phylogenetic analysis of antigen gene sequence variation within the *Neisseria meningitidis* clonal complex 4821 lineage, based on nucleotide sequences of the *NMB0315* gene. Red shading indicates the genomes with *NMB0315* allele 21; blue shading with *NMB0315* allele 194; green shading with *NMB0315* allele 335.

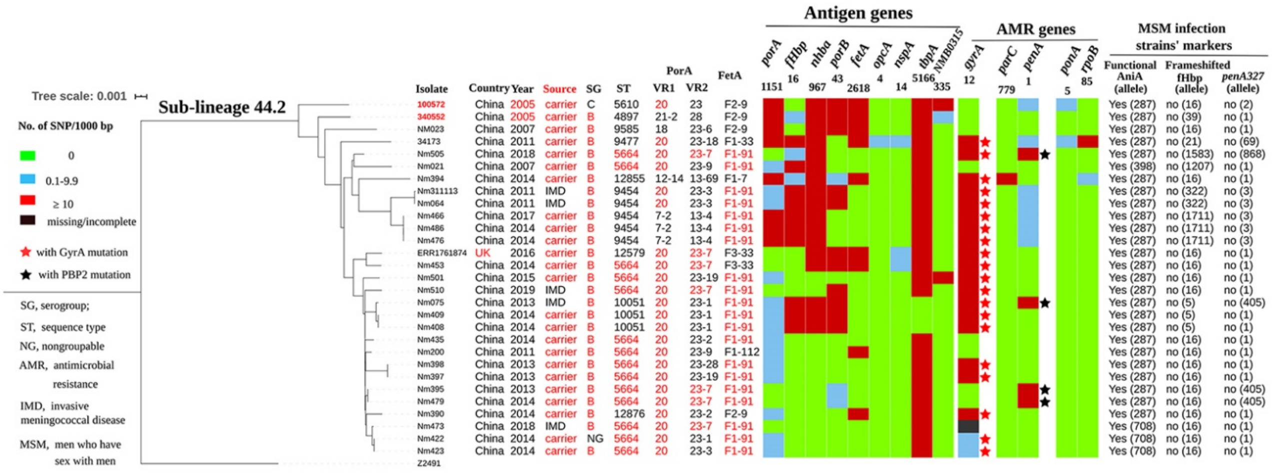

Appendix 2 Figure 16. Genetic diversity of sublineage 44.2 isolates. Red text indicates the oldest isolate of the sublineage in the columns of Isolate and Year, the isolates from countries outside of China in the Country column, and the dominant type/allele in the columns of Source, SG, ST, PorA-VR1, PorA-VR2, and FetA. The numbers underneath the antigen genes and AMR genes are the dominant alleles for that particular gene, and the colored blocks for SNPs/1,000 bp were determined using the allele number labeled above each column as the reference allele.

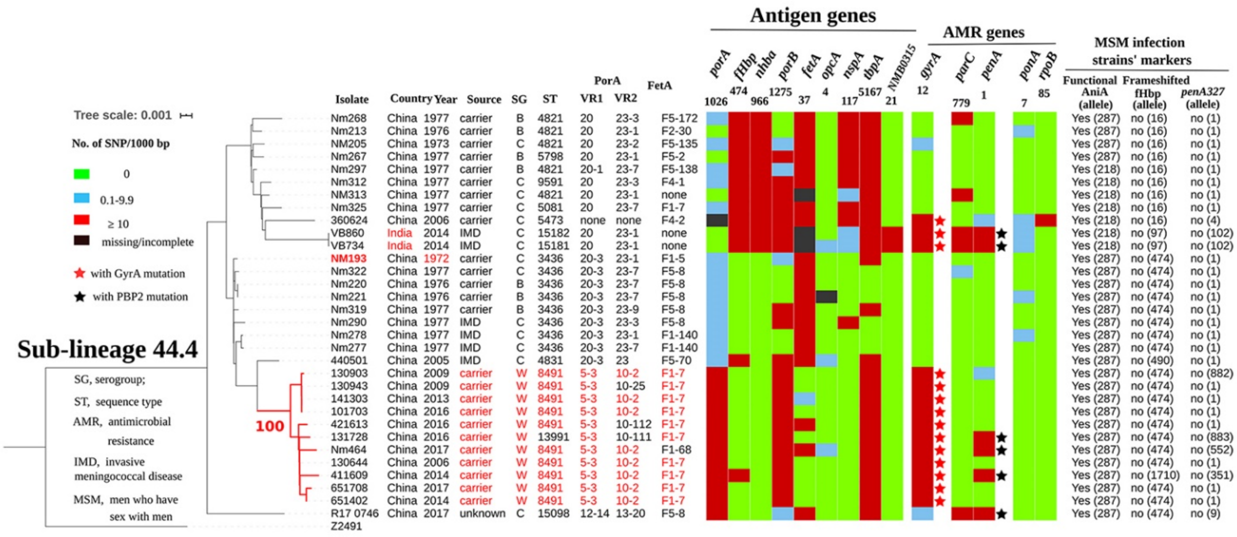

Appendix 2 Figure 17. Genetic diversity of sublineage 44.4 isolates. The red texts are used to indicate the oldest isolate of the sublineage in the columns of Isolate and Year, the isolates from countries outside of China in the Country column, and the dominant type/allele among serogroup W isolates in the columns of Source, SG, ST, PorA-VR1, PorA-VR2, and FetA. The numbers underneath the antigen genes and AMR genes are the dominant alleles for that particular gene, and the colored blocks for SNPs/1000 bp were determined using the allele number labeled above each column as the reference allele.

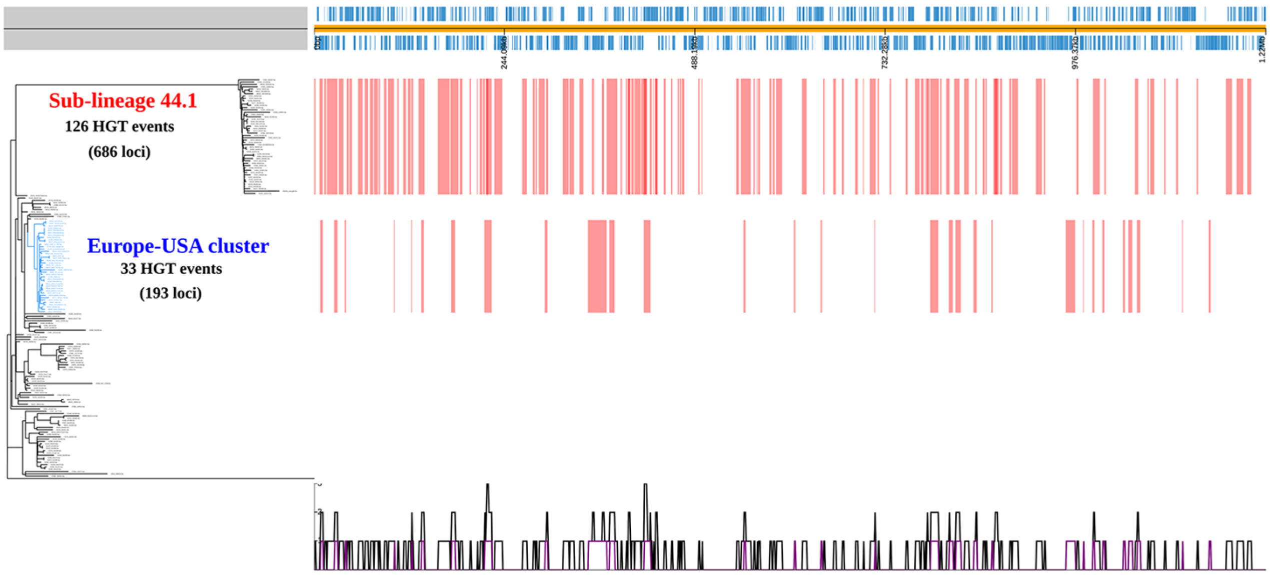

Appendix 2 Figure 18. Horizontal gene transfer (HGT) events predicted by Gubbins. Each red vertical line represents a HGT event. A total of 126 HGT events involved with 686 loci in L44.1; 33 events in Europe–USA cluster involved with 193 loci.
